# MICU1 and MICU2 Play an Essential Role in Mitochondrial Ca^2+^ Uptake, Growth, and Infectivity of the Human Pathogen Trypanosoma cruzi

**DOI:** 10.1128/mBio.00348-19

**Published:** 2019-05-07

**Authors:** Mayara S. Bertolini, Miguel A. Chiurillo, Noelia Lander, Anibal E. Vercesi, Roberto Docampo

**Affiliations:** a Departamento de Patologia Clínica, Faculdade de Ciências Médicas, Universidade Estadual de Campinas, Campinas, São Paulo, Brazil; b Center for Tropical and Emerging Global Diseases and Department of Cellular Biology, University of Georgia, Athens, Georgia, USA; Washington University School of Medicine

**Keywords:** MICU1, MICU2, Trypanosoma cruzi, calcium signaling, calcium uniporter, mitochondria

## Abstract

The mitochondrial Ca^2+^ uptake in trypanosomatids, which belong to the eukaryotic supergroup Excavata, shares biochemical characteristics with that of animals, which, together with fungi, belong to the supergroup Opisthokonta. However, the composition of the mitochondrial calcium uniporter (MCU) complex in trypanosomatids is quite peculiar, suggesting lineage-specific adaptations. In this work, we used Trypanosoma cruzi to study the role of orthologs for mitochondrial calcium uptake 1 (MICU1) and MICU2 in mitochondrial Ca^2+^ uptake. T. cruzi MICU1 (TcMICU1) and TcMICU2 have mitochondrial targeting signals, two canonical EF-hand calcium-binding domains, and localize to the mitochondria. Using the CRISPR/Cas9 system (i.e., clustered regularly interspaced short palindromic repeats with Cas9), we generated *TcMICU1* and *TcMICU2* knockout (-KO) cell lines. Ablation of either *TcMICU1* or *TcMICU2* showed a significantly reduced mitochondrial Ca^2+^ uptake in permeabilized epimastigotes without dissipation of the mitochondrial membrane potential or effects on the AMP/ATP ratio or citrate synthase activity. However, none of these proteins had a gatekeeper function at low cytosolic Ca^2+^ concentrations ([Ca^2+^]_cyt_), as occurs with their mammalian orthologs. *TcMICU1*-KO and *TcMICU2*-KO epimastigotes had a lower growth rate and impaired oxidative metabolism, while infective trypomastigotes have a reduced capacity to invade host cells and to replicate within them as amastigotes. The findings of this work, which is the first to study the role of MICU1 and MICU2 in organisms evolutionarily distant from animals, suggest that, although these components were probably present in the last eukaryotic common ancestor (LECA), they developed different roles during evolution of different eukaryotic supergroups. The work also provides new insights into the adaptations of trypanosomatids to their particular life styles.

## Abstract (precis)

Trypanosoma cruzi is the etiologic agent of Chagas disease and belongs to the early-branching eukaryotic supergroup Excavata. Its mitochondrial calcium uniporter (MCU) subunit shares similarity with the animal ortholog that was important to discover its encoding gene. In animal cells, the MICU1 and MICU2 proteins act as Ca^2+^ sensors and gatekeepers of the MCU, preventing Ca^2+^ uptake under resting conditions and favoring it at high cytosolic Ca^2+^ concentrations ([Ca^2+^]_cyt_). Using the CRISPR/Cas9 technique, we generated *TcMICU1* and *TcMICU2* knockout cell lines and showed that MICU1 and -2 do not act as gatekeepers at low [Ca^2+^]_cyt_ but are essential for normal growth, host cell invasion, and intracellular replication, revealing lineage-specific adaptations.

## INTRODUCTION

The mitochondrial calcium uniporter (MCU) complex mediates Ca^2+^ uptake from the cytosol into the mitochondrial matrix driven by the electrochemical gradient generated by the respiratory chain or ATP hydrolysis (1) and regulates mitochondrial metabolism (2), cytoplasmic Ca^2+^ signaling (3), and cell death (4).

The MCU complex of animal cells includes several components such as the membrane pore-forming subunit MCU (5, 6), its paralog and dominant-negative subunit, MCUb (7), the scaffolding subunit, mitochondrial calcium uniporter regulator 1 (MCUR1) (8), the essential mitochondrial regulator (EMRE) (9), and the proteins mitochondrial calcium uptake 1 (MICU1) (10), 2 (MICU2), and 3 (MICU3) (11).

Despite the large driving force for Ca^2+^ entry into the mitochondria, the intramitochondrial Ca^2+^ concentration ([Ca^2+^]_m_) is not different from that in the cytosol ([Ca^2+^]_cyt_) and several components of the MCU complex have been shown to have a role in regulating mitochondrial Ca^2+^ uptake. MICU1 was originally reported to act as a gatekeeper by inhibiting MCU-mediated mitochondrial Ca^2+^ uptake at low [Ca^2+^]_cyt_ (12, 13). MICU2, bound covalently to MICU1 through disulfide bridges, was subsequently reported to be the more relevant inhibitor (14). Further work reported the role of either MICU1 or MICU2 or both as gatekeepers in different cells (15–19). Finally, EMRE was proposed to have a role as a matrix Ca^2+^ sensor regulating Ca^2+^ influx (20).

Most of the studies on the MCU complex have been done with animal cells, while some structural studies of the recombinant MCU were done in fungi (21–24), a group of organisms that, together with animals, belong to the Opisthokonta supergroup of eukaryotes. However, the discovery of the molecular nature of MICU1 (10) and MCU (5, 6) was achieved thanks to the evolutionary conservation of the MCU complex in vertebrates (25) and trypanosomatids (26), which belong to the supergroup Excavata, and its absence in yeast (27, 28). Further studies of the MCU complex in trypanosomatids, which included the pathogenic parasites Trypanosoma cruzi, etiologic agent of Chagas disease, and Trypanosoma brucei, etiologic agent of African trypanosomiasis, or sleeping sickness, revealed significant differences from the animal MCU complex. Trypanosomes possess MCU, MCUb, MICU1, and MICU2 orthologs but lack MCUR1, EMRE, and MICU3 (29, 30). In contrast to what was reported in animal cells (7), MCUb is not a dominant-negative subunit but a Ca^2+^-conducting protein (31, 32). Trypanosomatids possess, in addition, two extra MCU paralogs that were named MCUc, and MCUd, which are also Ca^2+^-conducting subunits and form hetero-oligomers with MCU and MCUb (32).

In this work, we investigated whether T. cruzi MICU1 (TcMICU1) and TcMICU2 behave as the animal orthologs in modulating mitochondrial Ca^2+^ uptake. The results of this study indicate that both proteins are important for activating MCU, but do not form oligomers and do not behave as gatekeepers at low [Ca^2+^]_cyt_ as the animal orthologs do. Our results support the presence of these proteins in the last eukaryotic common ancestor (LECA) but the development of lineage-specific functions in the Excavata and Opisthokonta supergroups.

## RESULTS

### MICU1 and MICU2 homologs in Trypanosoma cruzi.

**FIG 1:**
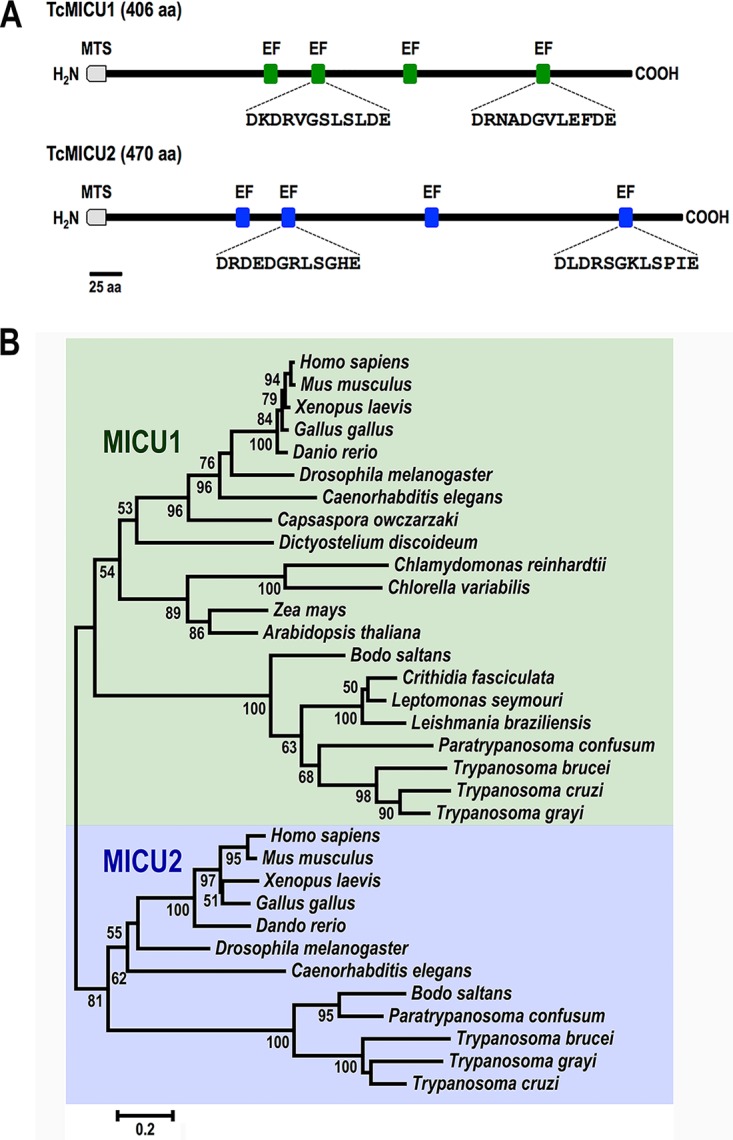
Structural domains and phylogeny of TcMCU1 and TcMICU2. (A) Domain structure of *TcMICU1* and *TcMICU2* highlighting the amino acid sequence of the two canonical EF-hands. (B) A phylogenetic tree was constructed using the neighbor-joining method with 33 selected MICU1 and MICU2 predicted homologs. The sequences branched into two divergent clades containing MICU1 and MICU2, which are enclosed in a green box and blue box, respectively. Bootstrap values higher than 50 are shown. The tree is drawn to scale, with branch lengths in the same units as those of the evolutionary distances used to infer the phylogenetic tree. The scale is in units of the number of amino acid substitutions per site. Accession numbers of sequences used for panels A and B are shown in Fig. S2.

Two genes encoding putative MICU1 and MICU2 proteins were identified in the T. cruzi genomic database (www.tritrypdb.org): *TcMICU1* (TcCLB.511391.210) and *TcMICU2* (TcCLB.510525.130) (29). The TcMICU1 and TcMICU2 predicted proteins have 406 and 468 amino acids, with estimated molecular masses of 46.7 and 53.2 kDa, respectively. A ClustalW amino acid sequence alignment showed that TcMICU1 and TcMICU2 share approximately 20% identity and 38% similarity. The predicted amino acid sequences of the TcMICU1 and TcMICU2 proteins display 22% and 23.9% overall sequence identity (44.4% and 40% of similarity), respectively, to their human orthologs. The prediction scores for the N-terminal mitochondrial targeting sequences, according to MitoProt II, were 0.86 and 0.76 for TcMICU1 and TcMICU2, respectively, suggesting their mitochondrial localization. Both predicted proteins have two canonical and two noncanonical EF-hand calcium-binding domains (Fig. 1A; see Fig. S1 in the supplemental material). A multiple-protein alignment was done using homologs from different organisms, including several kinetoplastids. The phylogenetic tree shows two main branches, including MICU1 and MICU2 homolog sequences (Fig. 1B). In turn, each of those two branches is divided into two others, one of them clustering orthologs from kinetoplastids. Interestingly, several genera of this evolutionary group, such as *Leptomonas*, *Crithidia*, and *Leishmania*, lack orthologs for MICU2. The amino acid sequences of the conserved canonical EF-hand domains of the MICU1 and MICU2 that were used for constructing the phylogenetic tree are shown in Fig. S2 in the supplemental material.

10.1128/mBio.00348-19.1 FIG S1 Alignments of human and Trypanosoma cruzi MICU1 (A) and MICU2 (B) protein sequences. Identical and similar residues are highlighted in dark and light blue, respectively. The predicted mitochondrial targeting signal is shown in orange. EF-hand domains are shown in magenta. In panel A, the polybasic sequence of MICU1 involved in the electrostatic interaction with the polyaspartate tail of EMRE (55) is enclosed in a green box. The putative DIME interacting domain (DID) of Homo sapiens MICU1 (HsMICU1) is shown in red. Vertical red arrows indicate two arginine residues of HsMICU1 involved in potential salt bridges with the D-ring of MCU. The alignment also shows basic residues in the C terminus of TcMICU1 that potentially could have this role (56). Download FIG S1, TIF file, 2.6 MB. Copyright © 2019 Bertolini et al. 2019 Bertolini et al. This content is distributed under the terms of the Creative Commons Attribution 4.0 International license.

10.1128/mBio.00348-19.2 FIG S2 Sequences used in Fig. 1. Protein IDs are from the NCBI database (https://www.ncbi.nlm.nih.gov), except those indicated with an asterisk, which were obtained from the TriTryp database (http://tritrypdb.org/). EF1 and EF2 represent EF-hand calcium-binding domains that were predicted with the highest scores according to PROSITE (http://prosite.expasy.org). #, the *Bodo saltans* MICU2 sequence in the database is incomplete: as a consequence, the first EF-hand is absent. Download FIG S2, TIF file, 2.1 MB. Copyright © 2019 Bertolini et al. 2019 Bertolini et al. This content is distributed under the terms of the Creative Commons Attribution 4.0 International license.

### Mitochondrial localization of *TcMICU1* and *TcMICU2* and effects of their overexpression.

**FIG 2:**
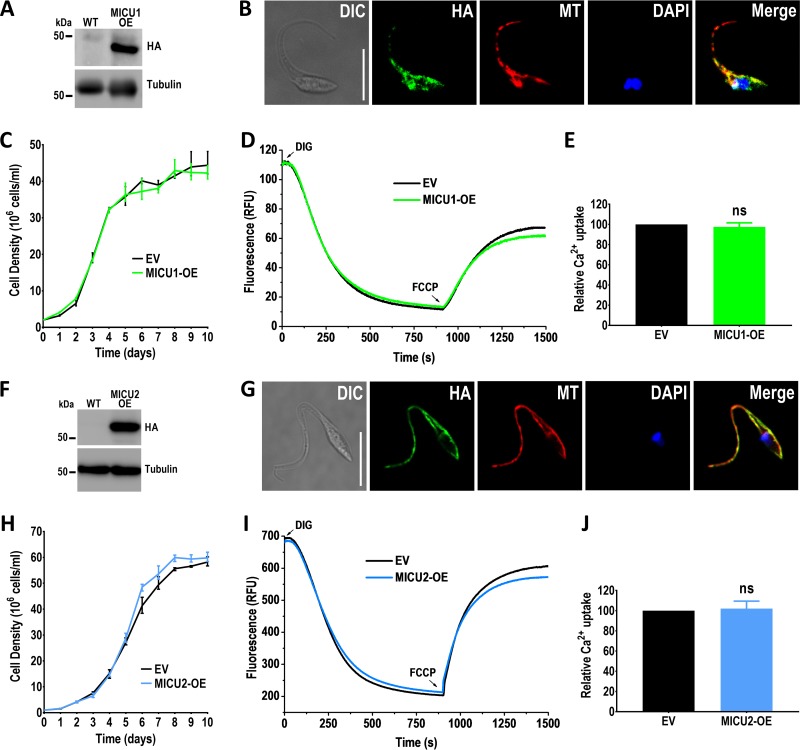
Localization of TcMICU1 and TcMICU2 and analysis of overexpression mutants. (A) TcMICU1-2×HA overexpression (MICU1-OE) was confirmed by Western blot analysis using anti-HA monoclonal antibodies, using wild-type (WT) cells as a control cell line. Tubulin was used as a loading control. (B) Immunofluorescence analysis (IFA) showed partial colocalization between TcMICU1-2×HA (HA [green]), and Mitotracker (MT [red]) in the merged image (yellow). DAPI is in blue. DIC, differential interference contrast. (C) Growth of control (epimastigotes transfected with pTREX-n empty vector [EV]) and MICU1-OE epimastigotes in LIT medium. (D) Representative traces of Ca^2+^ uptake by digitonin-permeabilized MICU1-OE and control epimastigotes in relative fluorescence units (RFU). The reaction was started after addition of 50 μM digitonin (DIG) in the presence of 20 μM free Ca^2+^, 5 mM succinate, and 0.5 μM Calcium Green-5N probe. Where indicated, 4 μM FCCP was added. Fluorescence changes were monitored using the Hitachi F-4500 spectrofluorometer. (E) Quantification of data from three experiments like that represented in panel D. (F) Western blot analysis of total protein extracts of WT and *TcMICU2*-2×HA (MICU2-OE) epimastigotes, using anti-HA antibodies. Tubulin was used as a loading control. (G) IFA showed partial colocalization between TcMICU2-2×HA (HA [green]), and Mitotracker (MT [red]) in the merged image (yellow). DAPI staining is shown in blue. (H) Growth of control (EV), and MICU2-OE epimastigotes in LIT medium. (I) Representative traces of Ca^2+^ uptake by digitonin-permeabilized MICU2-OE and control (EV) epimastigotes in RFU. The reaction was started after adding 50 μM DIG with the same experimental conditions and additions as in panel D. Fluorescence changes were monitored using the Hitachi F-7000 spectrofluorometer. (J) Quantification of data from three experiments like that represented in panel I. In panels E and J, values are means ± SD (*n *=* *3). ns, no significant difference (Student's *t* test).

To determine the cellular localization of TcMICU1 and TcMICU2, we overexpressed (-OE) their hemagglutinin (HA)-tagged versions (*TcMICU1*-OE and *TcMICU2*-OE) in T. cruzi epimastigotes. Whereas *TcMICU2*-2×HA was overexpressed using the pTREX-n vector following standard procedures, to obtain the *TcMICU1*-overexpressing cell line, we transfected epimastigotes with a construct in which the gene is linked to the blasticidin-resistant gene (*Bsd*) via the 2A peptide sequence, in order to coexpress the epitope-tagged TcMICU1 protein with the selection marker. Western blot analyses of total epimastigote extracts showed protein bands of the expected sizes, 48.9 and 55.4 kDa, for TcMICU1 and TcMICU2, respectively (Fig. 2A and F). Mitochondrial localization of both HA-tagged proteins was confirmed by colocalization with the mitochondrial marker Mitotracker (Fig. 2B and G). *TcMICU1*-OE and *TcMICU2*-OE cells had the same growth rate as control cells transfected with the pTREX-n empty vector (Fig. 2C and H). We also carried out immunoblot analyses under reducing and nonreducing conditions. In both cases, we only detected the monomeric forms of TcMICU1 and TcMICU2, indicating that they do not form dimers, at least in our overexpressing cells (see Fig. S3 in the supplemental material).

10.1128/mBio.00348-19.3 FIG S3 Western blots of TcMICU1-OE and TcMICU2-OE under nonreducing conditions. To study if TcMICU1 and TcMICU2 form oligomers *in vivo* through disulfide bonds, total protein extracts of TcMICU1 (MICU1-OE) and TcMICU2 (MICU2-OE) overexpressing epimastigotes were subjected to Western blot analysis under reducing (with β-mercaptoethanol [A and B]) and nonreducing (without β-mercaptoethanol [C and D]) conditions. Expression of HA-tagged proteins was confirmed using anti-HA monoclonal antibodies. Tubulin was used as a loading control. Download FIG S3, TIF file, 1.4 MB. Copyright © 2019 Bertolini et al. 2019 Bertolini et al. This content is distributed under the terms of the Creative Commons Attribution 4.0 International license.

To determine the ability of the mitochondria of the *TcMICU1*-OE and *TcMICU2*-OE cell lines to take up Ca^2+^, we monitored changes in fluorescence of Calcium Green-5N in digitonin-permeabilized epimastigotes in the presence of succinate as the substrate (Fig. 2D and I). After cell permeabilization, and in the presence of 20 μM Ca^2+^, the decrease in fluorescence indicates that Ca^2+^ is taken up by energized mitochondria while the extramitochondrial Ca^2+^ concentration ([Ca^2+^]_ext_) decreases. Addition of the protonophore carbonylcyanide *p*-trifluoromethoxyphenylhydrazone (FCCP) leads to an increase of fluorescence due to depolarization of the mitochondrial membrane potential (Δψ_m_) and consequent Ca^2+^ release. There were no significant differences in mitochondrial Ca^2+^ uptake from either *TcMICU1*-OE or *TcMICU2*-OE epimastigotes compared with control cells (Fig. 2D, E, I, and J).

We also tested mitochondrial Ca^2+^ uptake in a range of extramitochondrial free Ca^2+^ concentrations between 0.8 and 50 μM, using Fluo-4 and Calcium Green-5N Ca^2+^-sensitive fluorophores in cells overexpressing either *TcMICU1* or *TcMICU2*. No significant difference in Ca^2+^ uptake rates or in the thresholds for MCU activation (see Fig. S4A, B, E, and F in the supplemental material) was detected between control cells and either *TcMICU1-*OE or *TcMICU2-*OE epimastigotes. Interestingly, the MCU gatekeeping threshold in T. cruzi epimastigotes was estimated to be between 800 nM and 1.5 μM [Ca^2+^]_ext_. We did not observe alterations in the mitochondrial membrane potential (Δψ_m_), evaluated by changes in safranin O fluorescence in digitonin-permeabilized epimastigotes of *TcMICU1*-OE and *TcMICU2*-OE cells compared to control cells (Fig. S4C, D, G, and H).

10.1128/mBio.00348-19.4 FIG S4 Analysis of mitochondrial Ca^2+^ uptake and mitochondrial membrane potential in *TcMICU1*-OE and *TcMICU2*-OE epimastigotes. (A) Quantification of Ca^2+^ uptake rates at low extramitochondrial (0.8 and 1.5 μM) estimated free Ca^2+^ of control EV (empty vector) and *TcMICU1*-OE (MICU1-OE) epimastigotes. (B) Quantification of Ca^2+^ uptake rates at high estimated [Ca^2+^]_ext_ (15, 25, and 50 μM) of control (EV) and MICU1-OE epimastigotes. In panels A and B, values are means ± SD (*n* = 3). ns, no significant differences (one-way ANOVA with multiple comparisons). (C) Changes in mitochondrial membrane potential (Δψ_m_) of digitonin-permeabilized epimastigotes as detected by changes in safranin O fluorescence in control (EV) and MICU1-OE epimastigotes. Cells (5 × 10^7^) were added to the reaction buffer (2 ml) containing 0.2% BSA, 5 mM succinate, 50 μM EGTA, and 5 μM safranin O. The reaction was started with 50 μM digitonin (DIG), and 250 μM ADP, 1.5 μM carboxyatractyloside (CAT), and 4 μM FCCP were added where indicated. (D) Changes in safranin fluorescence after addition of ADP from three experiments like that shown in panel C. Values are means ± SD (*n *=* *3). ns, no significant differences (Student’s *t* test). (E) Quantification of Ca^2+^ uptake rates at low estimated [Ca^2+^]_ext_ (0.8 and 1.5 μM) of control (EV) and MICU2-OE epimastigotes. (F) Quantification of Ca^2+^ uptake rates at high estimated [Ca^2+^]_ext_ (15, 25, and 50 μM) of control (EV) and MICU2-OE epimastigotes. In panels E and F, values are means ± SD (*n *=* *3). ns, no significant differences (one-way ANOVA with multiple comparisons). (G) Changes in mitochondrial membrane potential (Δψ_m_) of digitonin-permeabilized epimastigotes as detected by changes in safranin O fluorescence in empty vector (EV) and MICU2-OE epimastigotes. The experimental conditions are the same as in panel C. (H) Changes in safranin O fluorescence after addition of ADP from 3 experiments as that shown in panel G. Values are means ± SD (*n* = 3). ns, no significant differences (Student’s *t* test). Fluorometric assays were performed using either the Hitachi F-4500 (TcMICU1-OE) or F-7000 (TcMICU2-OE) spectrofluorometer. Download FIG S4, TIF file, 0.7 MB. Copyright © 2019 Bertolini et al. 2019 Bertolini et al. This content is distributed under the terms of the Creative Commons Attribution 4.0 International license.

### Generation of *TcMICU1*-KO and *TcMICU2*-KO mutants and phenotypic changes.

**FIG 3:**
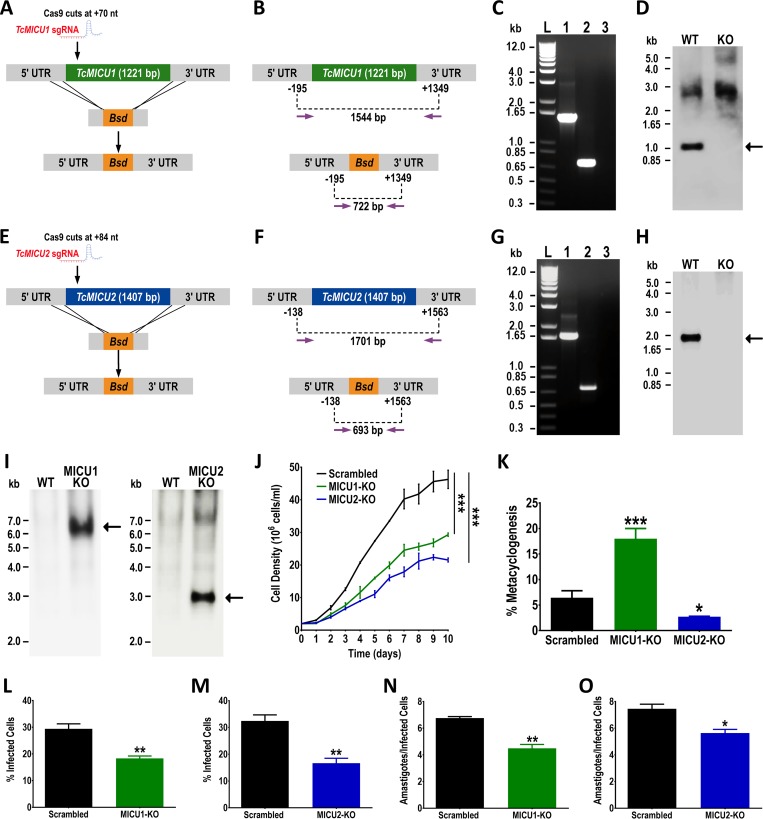
Phenotypic changes of *TcMICU1*-KO and *TcMICU2*-KO cells in different life cycle stages. (A) Schematic representation of the strategy used to generate a *TcMICU1-*KO mutant by CRISPR/Cas9-induced homologous recombination. A double-stranded gDNA break was produced by Cas9 at nt +70 of the *TcMICU1* ORF (1,221 bp). DNA was repaired with a blasticidin-S deaminase (*Bsd*) cassette containing 100-bp homologous regions from *TcMICU1* 5′ and 3′ untranslated regions (UTRs). (B) Primers (arrows) used to verify gene replacement by PCR. The intact locus generates a PCR product of 1,544 bp, while the disrupted locus generates a fragment of 722 bp. (C) PCR analysis showing that *TcMICU1* was ablated at its genomic locus and replaced in genomic DNA of the KO cell line. Lanes: L, 1-kb plus ladder; 1, wild type; 2, *TcMICU1-*KO; 3, PCR negative control. (D) Southern blot analysis of wild-type (WT) and *TcMICU1*-KO (KO) gDNA digested with PvuII restriction enzyme. The blot was hybridized with a biotin-labeled probe corresponding to 430 bp of *TcMICU1* (nt +655 to +1085). (E) Schematic representation of the strategy used to generate a *TcMICU2-*KO mutant by CRISPR/Cas9-induced homologous recombination. A double-stranded gDNA break was produced by Cas9 at nt +84 of the *TcMICU2* ORF (1,407 bp). DNA was repaired with a *Bsd* cassette containing 100-bp homologous regions from *TcMICU2* 5′ and 3′ UTRs. (F) Primers (arrows) used to verify gene replacement by PCR. The intact locus generates a PCR product of 1,701 bp, while the disrupted locus generates a fragment of 693 bp. (G) PCR analysis showing that *TcMICU2* was ablated at its genomic locus and replaced in genomic DNA of the KO cell line. Lanes: L, 1-kb plus ladder; 1, wild type; 2, *TcMICU2-*KO; 3, PCR negative control. (H) Southern blot analysis of WT and *TcMICU2*-KO (KO) gDNA digested with HindIII restriction enzyme. The blot was hybridized with a biotin-labeled probe corresponding to 436 bp of *TcMICU2* (nt +195 to +631). (I) Southern blot analysis of wild-type (WT) and *TcMICU1*-KO (left panel) or *TcMICU2*-KO (right panel) gDNA digested with HindIII restriction enzyme. The blot was hybridized with a biotin-labeled probe corresponding to the entire *Bsd* gene. (J) Growth of control (scrambled), MICU1-KO and MICU2-KO epimastigotes in LIT medium. One-way ANOVA with multiple comparisons was applied to growth rates calculated from each growth curve (***, *P* < 0.001). (K) Percentage of metacyclic trypomastigotes in epimastigote cultures after incubation in TAU 3AAG medium. Differentiation of epimastigotes to metacyclic trypomastigotes was quantified by staining with DAPI to distinguish the position of the kinetoplast by fluorescence microscopy. Values are means ± SD (*n *=* *3). *, *P* < 0.05, and ***, *P* < 0.001, by one-way ANOVA with multiple comparisons. (L and M) *TcMICU1*-KO (L) and *TcMICU2*-KO (M) trypomastigote infection of Vero cells. (N and O) Effect of *TcMICU1* (N) and *TcMICU2* (O) knockout in amastigote replication after 48 h. In panels L, M, N, and O, values are means ± SD (*n *=* *3). *, *P* < 0.05, and **, *P* < 0.01, by Student's *t* test.

To further explore the role of *TcMICU1* and *TcMICU2*, we generated null (knockout [-KO]) mutants for these two genes (*TcMICU1*-KO and *TcMICU2*-KO) using the CRISPR/Cas9 method (i.e., clustered regularly interspaced short palindromic repeats with Cas9), which has been successfully adapted recently to T. cruzi (31, 33–35) (Fig. 3A, B, E, and F). As described in Materials and Methods, T. cruzi epimastigotes were transfected with specific molecular constructs for the constitutive expression of Cas9 nuclease and single guide RNA (sgRNA) to target *TcMICU1* or *TcMICU2* genes (Fig. 3A and E). After selection with G418 and blasticidin, we obtained clonal populations from these cell lines by limiting dilution. Using specific sets of primers (see Table S1 in the supplemental material), we confirmed by PCR that both *TcMICU1* and *TcMICU2* genes were ablated and replaced by the DNA donor cassette with the resistance marker at the specific loci (Fig. 3B, C, F, and G). Southern blot analyses confirmed that *TcMICU1* (Fig. 3D) and *TcM1CU2* (Fig. 3H) were absent in genomic DNA of the KO cell lines. When the blots were hybridized with a biotin-labeled probe corresponding to *Bsd* sequence, a band with the estimated HindIII restriction fragment size corresponding to the replacement of *TcMICU1* and *TcMICU2* by the resistance marker gene (6,263 and 2,921 bp for *TcMICU1*-KO and *TcMICU2*-KO, respectively) (Fig. 3I) was detected only in genomic DNA (gDNA) of KO cells.

10.1128/mBio.00348-19.7 TABLE S1 Oligonucleotides used in this work. Download Table S1, DOCX file, 0.1 MB. Copyright © 2019 Bertolini et al. 2019 Bertolini et al. This content is distributed under the terms of the Creative Commons Attribution 4.0 International license.

The growth rate of *TcMICU1*-KO and *TcMICU2*-KO epimastigotes in LIT (liver infusion tryptose) medium was significantly lower than that in the control cell line transfected with a scrambled sgRNA (Fig. 3J). Interestingly, our attempts to generate a *TcMICU1 TcMICU2* double KO cell linage did not result in viable cells. We then evaluated the ability of null mutant epimastigotes to differentiate into metacyclic trypomastigotes (metacyclogenesis). Cultivation of epimastigote forms is carry out in the highly nutritive LIT medium supplemented with 10% fetal bovine serum (FBS), but to induce metacyclogenesis *in vitro*, we used a chemically defined medium, TAU 3AAG, that mimics triatomine urine (TAU) medium (36). While *TcMICU1*-KO cells were able to differentiate to metacyclic trypomastigotes in a higher proportion than control cells, *TcMICU2*-KO epimastigotes showed significantly reduced metacyclogenesis (Fig. 3K). Moreover, the ability of trypomastigotes to infect host cells, as well as the replication of intracellular amastigotes, was significantly affected by knockout of either *TcMICU1* or *TcMICU2*, compared to control cells (Fig. 3L to O).

### Mitochondrial Ca^2+^ uptake in *TcMICU1*-KO and *TcMICU2*-KO cells.

**FIG 4:**
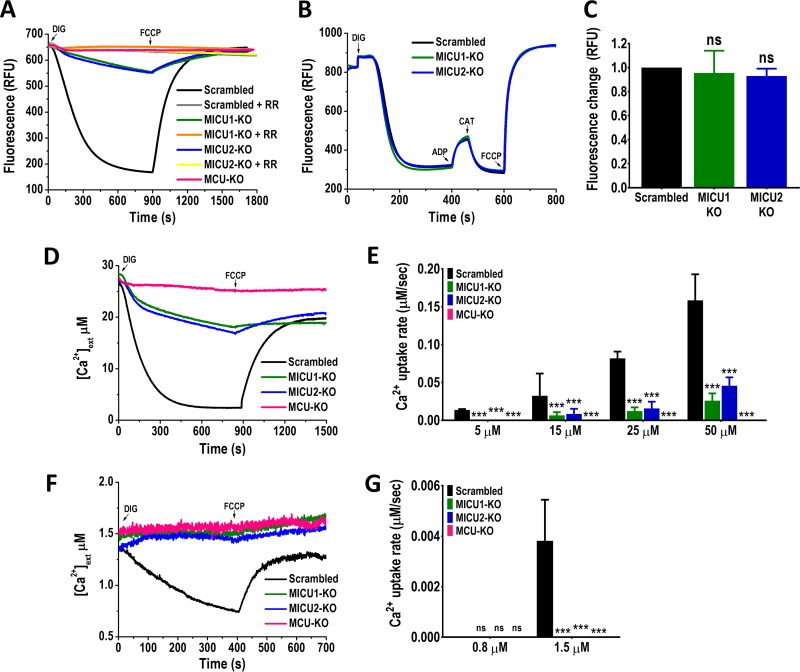
Analysis of mitochondrial Ca^2+^ uptake and membrane potential in *TcMICU1*-KO and *TcMICU2*-KO epimastigotes. (A) Representative Ca^2+^ uptake traces by digitonin-permeabilized epimastigotes in relative fluorescence units (RFU). Scrambled, scrambled control cells; MICU1-KO, *TcMICU1-*KO cells; MICU2-KO, *TcMICU2-*KO cells; MCU-KO, *TcMCU*-KO cells; RR, 5 μM ruthenium red (RR). The reaction was started after addition of 50 μM digitonin (DIG) in the presence of 20 μM CaCl_2_, 5 mM succinate, and 0.5 μM Calcium Green-5N probe. Where indicated, 4 μM FCCP was added. (B) Changes in mitochondrial membrane potential (Δψ_m_) of digitonin-permeabilized epimastigotes as detected by changes in safranin O fluorescence in scrambled, MICU1-KO, and MICU2-KO epimastigotes. Cells (5 × 10^7^) were added to the reaction buffer (2 ml) containing 0.2% BSA, 5 mM succinate, 50 μM EGTA and 5 μM safranin O. The reaction was started with 50 μM DIG, and 250 μM ADP, 1.5 μM carboxyatractyloside (CAT), and 4 μM FCCP were added where indicated. A decrease in fluorescence after permeabilization with digitonin indicated accumulation of the dye in energized mitochondria. Addition of ADP produced a small dissipation of membrane potential, indicating ADP phosphorylation. Δψ_m_ returned to its initial level after addition of the adenine nucleotide translocator inhibitor CAT. Addition of FCCP collapsed the membrane potential. (C) Changes in safranin O fluorescence after addition of ADP from three experiments like that shown in panel B. (D) Representative traces of Ca^2+^ uptake in digitonin-permeabilized epimastigotes at high [Ca^2+^]_ext_ (25 μM CaCl_2_) using Calcium Green-5N indicator in scrambled, MICU1-KO, and MICU2-KO cells. Other additions are as in panel A. (E) Quantification of Ca^2+^ uptake rates at high extramitochondrial (5, 15, 25, and 50 μM) estimated free Ca^2+^. (F) Representative traces of Ca^2+^ uptake in digitonin-permeabilized epimastigotes at low [Ca^2+^]_ext_ (1.5 μM CaCl_2_) using the Ca^2+^ indicator Fluo-4 in scrambled, MICU1-KO, and MICU2*-*KO epimastigotes. Other additions are as in panel A. (G) Quantification of Ca^2+^ uptake rates at low extramitochondrial (0.8 and 1.5 μM) estimated free Ca^2+^ concentrations. In panels C, E, and G, values are means ± SD (*n *=* *3). ns, no significant differences. ***, *P* < 0.001 by one-way ANOVA with multiple comparisons.

The capacity of mitochondria from digitonin-permeabilized *TcMICU1*-KO and *TcMICU2*-KO epimastigotes to take up Ca^2+^ was first evaluated using the Calcium Green-5N probe in the presence of 20 μM Ca^2+^ (Fig. 4A). Under these conditions, Ca^2+^ is taken up by mitochondria of control (scrambled) cells, but not of epimastigotes in which the MCU gene was ablated (*TcMCU*-KO) (31). Subsequent dissipation of Δψ_m_ by FCCP caused a rapid increase in fluorescence, indicating mitochondrial Ca^2+^ release. Both *TcMICU1*-KO and *TcMICU2*-KO epimastigotes displayed a reduced capacity for mitochondrial Ca^2+^ uptake (Fig. 4A). Mitochondrial Ca^2+^ transport of control and *TcMICU1*-KO or *TcMICU2*-KO epimastigotes was blocked by ruthenium red (5 μM), indicating that Ca^2+^ uptake was mediated by MCU.

To determine whether the defect in mitochondrial Ca^2+^ uptake in the null mutant cells was secondary to mitochondrial membrane depolarization, we measured the mitochondrial membrane potential (Δψ_m_) of digitonin-permeabilized epimastigotes, in the presence of succinate as a mitochondrial substrate, using safranin O. Knockout of either *TcMICU1* or *TcMICU2* did not affect the Δψ_m_ (Fig. 4B and C). As the assays using safranin O to evaluate Δψ_m_ were performed in the presence of bovine serum albumin (BSA), we also performed these experiments in the absence of BSA. BSA protects or restores mitochondrial functions by reducing the amount of free fatty acids, which can alter the mitochondrial surface charge, affecting the permeability to ions (37–39). We recorded changes in safranin O fluorescence and found no differences in Δψ_m_ at the steady state between *TcMICU1*-KO and *TcMICU2*-KO and control cells in the absence (see Fig. S5A and B in the supplemental material) or presence (Fig. S5C and D) of BSA in the reaction medium. In addition, we were not able to restore the capacity of permeabilized *TcMICU1*-KO and *TcMICU2*-KO epimastigotes to take up mitochondrial Ca^2+^ when assays were carried out in the presence of BSA in the reaction medium (Fig. S5E and F). Therefore, ablation of *TcMICU1* or *TcMICU2* results in a diminished ability of mitochondria to take up Ca^2+^ without affecting their Δψ_m_.

10.1128/mBio.00348-19.5 FIG S5 Analysis of mitochondrial membrane potential in *TcMICU1*-KO and *TcMICU2*-KO epimastigotes. (A) Changes in mitochondrial membrane potential (Δψ_m_) of digitonin-permeabilized epimastigotes as detected by changes in safranin O fluorescence in control (scrambled), *TcMICU1-*KO (MICU1-KO), and *TcMICU2-*KO (MICU2-KO) epimastigotes. Cells (5 × 10^7^) were added to the reaction buffer (2 ml) without BSA and with 5 mM succinate, 50 μM EGTA, and 5 μM safranin O. The reaction was started with 50 μM digitonin (DIG). (B) Changes in safranin O fluorescence after digitonin addition (between 100 and 200 s) in cells used in panel A. Values are means ± SD (*n *=* *3). ns, no significant differences (one-way ANOVA with multiple comparisons). (C) Changes in mitochondrial membrane potential (Δψ_m_) of digitonin-permeabilized epimastigotes as detected by changes in safranin O fluorescence in scrambled, MICU1-KO, and MICU2-KO epimastigotes. Cells (5 × 10^7^) were added to the reaction buffer (2 ml) with 0.2% BSA, 5 mM succinate, 50 μM EGTA, and 5 μM safranin O. The reaction was started with 50 μM DIG. (D) Changes in safranin fluorescence after digitonin addition (between 100 and 200 s) in cells used in panel C. Values are means ± SD (*n *=* *3). ns, no significant differences (one-way ANOVA with multiple comparisons). (E) Representative traces of Ca^2+^ uptake by digitonin-permeabilized scrambled, MICU1-KO, MICU2-KO, and MCU-KO epimastigotes, expressed in relative fluorescence units (RFU). The reaction was started after adding 50 μM DIG in the presence of 20 μM free Ca^2+^, 5 mM succinate, 0.2% BSA, and 0.5 μM Calcium Green-5N probe. (F) Quantification of data from experiments like that shown in panel E. Values are means ± SD (*n *=* *3). ***, *P* < 0.001 by one-way ANOVA with multiple comparisons. ns, no significant differences. Download FIG S5, TIF file, 0.6 MB. Copyright © 2019 Bertolini et al. 2019 Bertolini et al. This content is distributed under the terms of the Creative Commons Attribution 4.0 International license.

**FIG 5:**
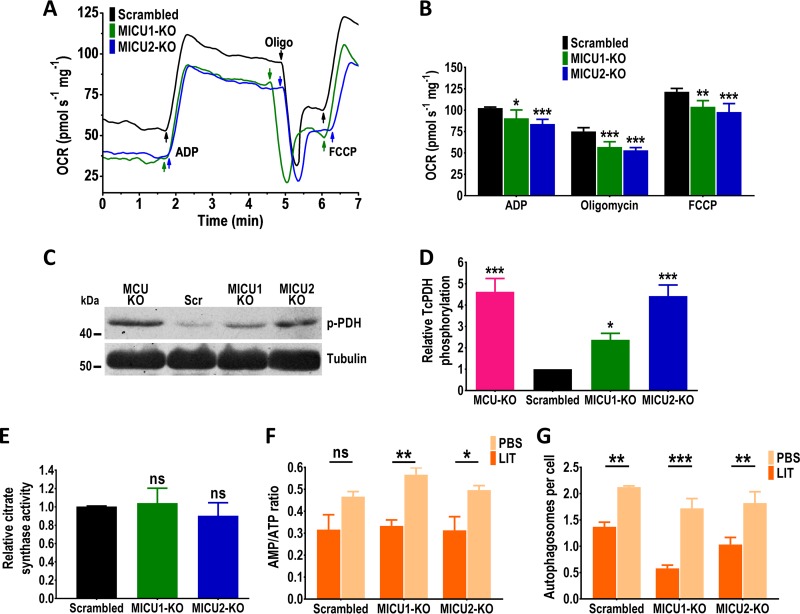
Mitochondrial and autophagy changes in *TcMICU1*-KO and *TcMICU2*-KO epimastigotes. (A) Representative traces of oxygen consumption rate (OCR) by digitonin-permeabilized control (scrambled), *TcMICU1-*KO (MICU1-KO), and *TcMICU2-*KO (MICU2-KO) epimastigotes. (B) OCR of digitonin-permeabilized scrambled, MICU1-KO, and MICU2-KO cells. Bar charts show OCR after addition of 100 μM ADP (respiration stimulated by oxidative phosphorylation [OXPHOS]), 1 μg/ml oligomycin (minimal respiratory rate), and 1 μM FCCP (maximal respiratory capacity). Values are means ± SD (*n *=* *3). *, *P* < 0.05, **, *P* < 0.01, and ***, *P* < 0.001, by two-way ANOVA with Dunnett’s multiple comparisons. (C) Representative Western blot of TcPDH-E1α subunit phosphorylation in *TcMCU*-KO, scrambled (Scr), *TcMICU1*-KO, and *TcMICU2*-KO cell lines. The antibody used detects the T. cruzi PDH-E1α subunit phosphorylated protein (expected size of 42.8 kDa). Tubulin was used as a loading control. (D) Densitometry analysis of three Western blots (as in panel C). Densitometric analysis was performed with ImageJ v1.48 software. Values are mean ± SD (*n *=* *3). *, *P* < 0.05, and ***, *P* < 0.001, by one-way ANOVA with Dunnett’s multiple-comparison test. (E) Relative citrate synthase activity of scrambled, MICU1-KO, and MICU2-KO epimastigotes. Values are means ± SD (*n *=* *3). ns, no significant differences (one-way ANOVA with multiple comparisons). (F) AMP/ATP ratios of control (scrambled), *TcMICU1*-KO, and *TcMICU2*-KO cells incubated in LIT medium or PBS for 16 h. (G) Number of autophagosomes per cell observed by fluorescence microscopy images of scrambled, *TcMICU1*-KO, and *TcMICU2*-KO epimastigotes labeled with anti-TcATG8.1 antibody after incubation in LIT medium or PBS for 16 h. In panels F and G, values are means ± SD (*n *=* *3). *, *P* < 0.05, **, *P* < 0.01, and ***, *P* < 0.001, by two-way ANOVA with Sidak’s multiple-comparison test.

We next examined whether ablation of *TcMICU1* and *TcMICU2* affected Ca^2+^ uptake in the same way at low and high [Ca^2+^]_ext_ (Fig. 4D to G). When mitochondrial Ca^2+^ uptake was measured at high [Ca^2+^]_ext_ (5 to 50 μM), a similar significant reduction of Ca^2+^ uptake rate in the null mutants compared with control cells was observed (Fig. 4D and E). However, *TcMICU1*-KO and *TcMICU2*-KO cells did not show mitochondrial Ca^2+^ uptake at a [Ca^2+^]_ext_ of 1.5 μM (Fig. 4F and G) or 5 μM (Fig. 4E). These results are in contrast to those obtained with animal mitochondria, where ablation of either MICU1 or MICU2 determined enhanced mitochondrial Ca^2^*^+^* uptake at low [Ca^2+^]_ext_. At lower [Ca^2+^]_ext_ (0.8 μM), we were not able to detect mitochondrial Ca^2+^ uptake by either control or mutant cells (Fig. 4G), results that are compatible with the buffering characteristics of mitochondria and a “set point” for Ca^2+^ uptake between 0.8 and 1.5 μM (40).

### Respiration and autophagy in *TcMICU1*-KO and *TcMICU2*-KO cells.

To study the effects of *TcMICU1* and *TcMICU2* ablation on cell bioenergetics, we measured the mitochondrial oxygen consumption rate (OCR) (Fig. 5A) under basal (state 2), ADP-stimulated (state 3), oligomycin-inhibited (state 4), and FCCP-stimulated (state 3u) conditions in control (scrambled) and mutant digitonin-permeabilized cells in the presence of succinate as the substrate. Control and mutant mitochondria showed well-coupled respiration, although OCRs in the presence of ADP, oligomycin, and FCCP were significantly lower in both *TcMICU1*-KO and *TcMICU2*-KO mitochondria (Fig. 5B). Respiratory control rates (state 3/4) were 1.38 ± 0.09, 1.54 ± 0.08, and 1.54 ± 0.09 for control, *TcMICU1*-KO, and *TcMICU2*-KO cells, respectively (*n *=* *3). Then we proceeded to analyze the level of pyruvate dehydrogenase (PDH) phosphorylation in these parasites by Western blotting as described previously (33). Our results indicate that T. cruzi PDH (TcPDH) phosphorylation in *TcMICU1*-KO and *TcMICU2-*KO epimastigotes is significantly higher than in control cells (scrambled) (Fig. 5C and D). We included in this experiment *TcMCU*-KO cells, which also exhibited an increased level of PDH phosphorylation (33). These results suggest that in *TcMICU1*-KO and *TcMICU2*-KO cells, the reduced capacity of mitochondria to take up Ca^2+^ determines a low activity of Ca^2+^-sensitive TcPDH phosphatase (TcPDP) and in consequence an increase in the inactive form (phosphorylated) of TcPDH. We also measured citrate synthase activity as an indicator of mitochondrial integrity (33, 41) and found no differences in *TcMICU1*-KO and *TcMICU2*-KO compared to control cells (Fig. 5E). Finally, we evaluated differences in energy levels (AMP/ATP ratio) and autophagy of control and KO cells incubated in LIT medium and under starvation conditions (phosphate-buffered saline [PBS]), as described before (31, 33) (Fig. 5F and G). We observed a significantly higher AMP/ATP ratio under starvation conditions in both *TcMICU1*-KO and *TcMICU2*-KO cells, although there were no significant differences compared with control cells (Fig. 5F). Moreover, we used antibodies against ATG8.1 autophagy marker Atg8.1, which is an ortholog of LC3-II in mammalian cells (42), to assess autophagy as previously described in T. cruzi (31, 33, 43), and we observed an increased number of autophagosomes per cell in control, *TcMICU1*-KO, and *TcMICU2*-KO cells incubated under starvation conditions compared with cells cultured in LIT medium. However, no differences were found comparing *TcMICU1*-KO and *TcMICU2*-KO with control cells (Fig. 5G).

## DISCUSSION

We report that ablation of *TcMICU1* or *TcMICU2* significantly reduces the mitochondrial capacity to take up Ca^2+^ and increases the [Ca^2+^] required for MCU activation without affecting Δψ_m_, suggesting a role in Ca^2+^ sensing. Both proteins are important for normal growth of epimastigotes in LIT culture medium, for differentiation to metacyclic trypomastigotes, for *in vitro* trypomastigote invasion of host cells, and for intracellular replication of amastigotes. *TcMICU1*-KO and *TcMICU2*-KO cells show a lower oxygen consumption rate compared with control epimastigotes. A normal citrate synthase activity indicates that there is no reduction in mitochondrial mass or content, as previously observed in *TcMCUb*-KO cells (31). Finally, we were not able to obtain stable *TcMICU1 TcMICU2* double knockout mutants, suggesting that, different from those reported in mammalian cell lines (19), the absence of both genes has serious adverse effects on T. cruzi survival.

Searches of genome and protein databases suggest that all kinetoplastid protists have orthologs for MICU1. However, we only found orthologs for MICU2 in the ancestral free-living bodonid Bodo saltans, the early-branched trypanosomatid Paratrypanosoma confusum, and species of the genus *Trypanosoma*, which includes pathogens such as T. cruzi, T. brucei, and Trypanosoma congolense. Evolutionarily, all these kinetoplastid species represent the transition from a free-living to a parasitic lifestyle (44, 45). It has been suggested that *MICU1* paralogs derived from a gene duplication event prior to the appearance of vertebrates (11). Our phylogenetic analysis suggests that *MICU2* was lost subsequent to the divergence of this branch, as occurred with *MICU1* in fungi (46).

In contrast to the results observed in HeLa cells, where overexpression of *MICU1* or *MICU2* resulted in marked enhancement or decrease, respectively, of mitochondrial Ca^2+^ accumulation (14), we did not detect changes in mitochondrial Ca^2+^ uptake by permeabilized epimastigotes when either *TcMICU1* or *TcMICU2* was overexpressed. Overexpression of either *TcMICU1* or *TcMICU2* did not affect growth of epimastigotes. Also, in contrast to what happens in mammalian cells, where MICU1 and MICU2 apparently play nonredundant roles in the regulation of the MCU complex (47), ablation of *TcMICU1* or *TcMICU2* gave rise to very similar outcomes in terms of their impact on mitochondrial Ca^2+^ uptake and in most of the phenotypic changes studied. Notably, it has been demonstrated that the loss of MICU1 in vertebrates leads to a concomitant loss of MICU2 and led to the suggestion that the loss of MCU gatekeeping in MICU1 knockdown cells is caused by the loss of MICU2 (11, 14, 48). Furthermore, *MICU2*^−/−^ mice had significantly reduced protein levels of MCU and MICU1, suggesting that the stability of the MCU complex may depend on the interaction among different components (49).

The absence of TcMICU1 or TcMICU2 rendered mitochondria significantly less efficient to take up Ca^2+^ across the entire range of Ca^2+^ concentrations evaluated (800 nM to 50 μM). In addition, the threshold for Ca^2+^ uptake was affected by loss of either TcMICU1 or TcMICU2, as indicated by impaired uptake when given a [Ca^2+^]_ext_ below the apparent control threshold. While permeabilized control cells were able to take up Ca^2+^ when the extramitochondrial Ca^2+^ concentration was between 800 nM and 1.5 μM, for permeabilized *TcMICU1*-KO and *TcMICU2*-KO epimastigotes, 5 μM [Ca^2+^]_ext_ was still not sufficient to stimulate Ca^2+^ uptake. It has been reported that the mitochondria of permeabilized epimastigotes (40, 50) or amastigotes (51) are able to accumulate Ca^2+^ up to a set point of 700 to 900 nM Ca^2+^, which is in the range of the threshold for mitochondrial Ca^2+^ uptake observed in control permeabilized epimastigotes in this work. Contrary to our results in T. cruzi, loss-of-function studies in vertebrates indicated that loss of either MICU1 or MICU2 altered the threshold for mitochondrial Ca^2+^ uptake, which occurs at [Ca^2+^]_cyt_ below that of wild-type cells (18, 19, 48). Therefore, MICU1-MICU2 dimers would function as gatekeepers of the MCU in animal cells, and by setting the [Ca^2+^]_cyt_ threshold for MCU activation, Ca^2+^ uptake only occurs at high [Ca^2+^]_cyt_ (12, 13, 16, 18, 52). Our results showing elevated phosphorylation of the TcPDH-E1α subunit in *TcMICU1*-KO or *TcMICU2*-KO cells is in contrast to what occurs in mammalian cells, where basal [Ca^2+^]_m_ is constitutively elevated when MICU1 expression is downregulated (12).

We investigated whether *TcMICU1* or *TcMICU2* genes form oligomeric complexes through covalent disulfide bonds as described for the mammalian orthologs (14, 53). However, despite the presence of cysteine residues at the C-terminal end of the proteins, immunoblot analyses of total protein extracts of *TcMICU1-*OE or *TcMICU2-*OE epimastigotes carried out under reducing and nonreducing conditions showed only the predicted bands for the monomeric forms of the proteins. Recently, MICU1-MICU2 heterodimers stabilized by disulfide bonds were shown to establish a cooperative interaction with Ca^2+^ to function as on/off switch to fine-tune the MCU and thus respond to changes in [Ca^2+^]_ext_ (19). Moreover, pulldown and coimmunoprecipitation assays of human MICU1 and MICU2 proteins suggested that, in addition to disulfide bonds, salt bridges created by Arg221 in MICU1 and Asp330 in MICU2 also contribute to MICU1-MICU2 heterodimer formation (54).

Consistent with the absence of an EMRE ortholog in the T. cruzi genome, the predicted sequence of TcMICU1 lacks the polybasic sequence (KKKKR) that has been reported to bind the polyaspartate tail of EMRE by electrostatic interactions (55). Alternatively, a direct interaction between TcMICU1 and the selectivity filter domain of MCU, as described recently for mammalian cells (56, 57), could exist to regulate mitochondrial Ca^2+^ uptake in T. cruzi (Fig. S1A).

The role of mitochondrial Ca^2+^ in T. cruzi has been recently studied in a knockout cell line for the Ca^2+^-sensitive pyruvate dehydrogenase phosphatase (*TcPDP*) gene (33). *TcPDP-KO* cells showed phenotypic features similar to those we found here for *TcMICU1*-KO or *TcMICU2*-KO cells, such as slower growth, reduced infectivity, and lower oxygen consumption rate. Loss of *TcPDP* resulted in increased levels of phosphorylated pyruvate dehydrogenase, which is the inactive form, suggesting a disruption of the link between glycolysis and the citric acid cycle. Therefore, a significant reduction in mitochondrial Ca^2+^ uptake would lead to inactivation of intramitochondrial dehydrogenases affecting energy metabolism.

In summary, our results indicate that the EF-hand-containing proteins TcMICU1 and TcMICU2 are essential components of the MCU complex of T. cruzi. TcMICU1 and TcMICU2 have significantly different properties from those of their animal orthologs: (i) they seem to have redundant roles, unless the loss of one of them could affect the other, (ii) they apparently do not form dimers linked by disulfide bonds, (iii) their overexpression does not affect mitochondrial Ca^2+^ uptake, (iv) their absence significantly decreases mitochondrial Ca^2+^ uptake and increases the [Ca^2+^]_ext_ set point needed for Ca^2+^ uptake, suggesting that they could not function as gatekeepers but rather in stabilization of the MCU complex, and (v) their ablation results in alterations in the oxidative metabolism of the parasites, probably resulting in decreased replication, as either epimastigotes or amastigotes, and decreased host invasion by trypomastigotes. Our results suggest that although MICU1 and MICU2 were present in LECA, they develop different lineage specific properties in the Excavata and Opithokonta supergroups.

## MATERIALS AND METHODS

### Chemicals and reagents.

Rabbit polyclonal antibody against TcATG8.1 was a gift from Vanina Alvarez (Universidad Nacional de San Martin, Argentina). High-fidelity Platinum *Taq* DNA polymerase, Calcium Green-5N, Fluo-4, MitoTracker deep red FM, Alexa-conjugated secondary antibodies, an ATP determination kit, Pierce ECL (enhanced chemiluminescence) Western blotting substrate, a bicinchoninic acid (BCA) protein assay kit, the North2South biotin random prime labeling kit, the North2South chemiluminescent hybridization and detection kit, and HA epitope tag monoclonal antibody were from Thermo Fisher Scientific (Waltham, MA). Blasticidin S HCl, the BenchMark prestained protein ladder, the BenchMark protein ladder, Alexa Fluor-conjugated secondary antibodies, and anti-mouse horseradish peroxidase (HRP)-conjugated secondary antibodies were purchased from Life Technologies (Grand Island, NY). The Wizard Plus SV miniprep purification system, Wizard SV gel and PCR cleanup system, GoTaq DNA polymerase, and T4 DNA ligase were from Promega (Madison, WI). Anti-serine 293 antibodies to phosphorylated PDH-E1α were from Abcam (Cambridge, MA). Antarctic phosphatase, restriction enzymes, and Q5 high-fidelity DNA polymerase were from New England Biolabs (Ipswich, MA). Fluoromount-G was from SouthernBiotech (Birmingham, AL). DNA oligonucleotides were purchased from Exxtend Biotecnologia, Ltd. (Campinas, Brazil). The protein assay reagent, Precision Plus protein dual color standards, anti-rabbit HRP-conjugated secondary antibodies, and nitrocellulose membranes were from Bio-Rad (Hercules, CA). Anti-c-Myc monoclonal antibody (clone 9E10) was from Santa Cruz Biotechnology (Dallas, TX). Antitubulin monoclonal antibody, carboxyatractyloside (CAT), oligomycin, safranin O, carbonylcyanide *p*-trifluoromethoxyphenylhydrazone (FCCP), Benzonase nuclease, puromycin, G418, mammalian cell protease inhibitor cocktail (Sigma P8340), other protease inhibitors, and all other reagents of analytical grade were from Sigma (St. Louis, MO).

### Culture methods.

T. cruzi epimastigotes (Y strain) were grown in liver infusion tryptose (LIT) medium (5.4 mM KCl, 150 mM NaCl, 24 mM glucose, 5% [vol/vol] liver extract, 0.02% [wt/vol] hemin, 2% [wt/vol] yeast extract, 1.5% [wt/vol] tryptose) (58) containing 10% heat-inactivated fetal bovine serum (FBS) at 28°C. Mutant cell lines were maintained in medium containing 250 μg/ml G418, 10 μg/ml blasticidin, or 5 μg/ml puromycin. The growth rate of epimastigotes was determined by counting cells in a Muse cell analyzer or in a Neubauer chamber. Tissue culture cell-derived trypomastigotes were obtained from Vero cells infected with metacyclic trypomastigotes as described below. T. cruzi trypomastigote forms were collected from the culture medium of infected host cells, using a modification of the method of Schmatz and Murray (59) as described previously (60). Vero cells were grown in RPMI supplemented with 10% fetal bovine serum and maintained at 37°C with 5% CO_2_.

### Sequence analysis.

Molecular constructs were verified by sequencing at the LaCTAD facility (http://www.lactad.unicamp.br) or Helixxa facility (http://www.helixxa.com.br). Primer design and sequence analysis were carried out using DNAMAN software (version 7.212; Lynnon Corp., Canada).

### *In silico* analyses.

Putative *MICU1* and *MICU2* genes were identified in the T. cruzi genome database (http://www.tritrypdb.org) (61). Both predicted amino acid sequences were aligned to 33 selected MICU1 and MICU2 orthologs obtained from the tritrypdb.org and NCBI (https://www.ncbi.nlm.nih.gov) databases using the ClustalW method in MEGA7 (62) software. Evolutionary analyses were conducted in MEGA7 (62) using the neighbor-joining method (63) and the bootstrap method with 1,000 replicates (64). The evolutionary distances were computed using the JTT matrix-based method of Jones et al. (65). The rate variation among sites was modeled with a gamma distribution (shape parameter = 4). MitoProt II (66) and PROSITE (67) were used to predict mitochondrial targeting sequence and EF-hand domains, respectively.

### *TcMICU1* and *TcMICU2* overexpression.

To enhance the expression efficiency of *TcMICU1* in T. cruzi epimastigotes, we generated a version of this gene linked with *Bsd* to the C terminus via a P2A peptide sequence. The full-length *TcMICU1* sequence from the T. cruzi Y strain (1,221 nucleotides [nt]), tagged with 2×HA, was previously cloned into pTREX-n vector. Subsequently, *TcMICU1*-2×HA fragment was amplified using primers 1 and 2 (Table S1) using pTREX-n-*TcMICU1*-2×HA as the template, while P2A-*Bsd* fusion was obtained with primers 3 and 4 (Table S1) using plasmid LwCas13a-msfGFP-2A-BLAST as the template (68). Then, we used these two fragments as the template in an overlap PCR, including primers 1 and 4 (Table S1). Finally, *TcMICU1*-2×HA-P2A-*Bsd* was cloned into pTREX-n vector by XbaI/XhoI restriction sites. The *TcMICU2* open reading frame (ORF [1,427 nt]) was PCR amplified using a reverse primer that includes a 2×HA epitope tag coding sequence (primers 5 and 6 [Table S1]), resulting in a final sequence of 1,467 nt. This sequence was cloned into the pTREX-n vector by restriction sites EcoRI/XhoI and subsequently used to transfect T. cruzi epimastigotes (31). Gene cloning was confirmed by PCR and sequencing. *TcMICU1* and *TcMICU2* overexpression was confirmed by Western blot analysis using anti-HA antibodies.

### Knockout of *TcMICU1*.

Chimera single guide RNA (sgRNA) sequences to target the *TcMICU1* gene (TryTripDB identifier [ID] TcCLB.511391.210) were PCR amplified (primers 7 and 8 [Table S1]) from plasmid pUC_sgRNA, as previously described (34). Selection of the protospacer was performed using EuPaGDT (Eukaryotic Pathogen CRISPR guide RNA Design Tool [http://grna.ctegd.uga.edu/]). The protospacer sequence was included into the forward primer, while a common reverse primer was used for sgRNA amplification. These primers also contained a BamHI restriction site for cloning into Cas9/pTREX-n (34) to generate the *TcMICU1-*sgRNA/Cas9/pTREX-n construct. The sgRNA orientation was verified by PCR using the specific TcMICU1-sgRNA forward primer and the HX1 reverse primer (primers 7 and 9 [Table S1]) (34). Positive clones that generate a 190-bp PCR fragment were also sequenced. A scrambled sgRNA (Scr-sgRNA/Cas9/pTREX-n) was used as a control. A DNA donor cassette designed to promote homologous directed repair and replacement of the *TcMICU1* ORF was obtained by PCR using a set of long primers (ultramers) containing 120 nucleotides, from which 100 nucleotides correspond to the first 100 nt (forward ultramer) and the last 100 nt (reverse ultramer) of *TcMICU1* ORF, with 20 nt annealing on blasticidin-S deaminase (*Bsd*) gene (primers 10 and 11 [Table S1]). The *TcMICU1-*sgRNA/Cas9/pTREX-n construct and linear *Bsd* cassette were used to cotransfect T. cruzi epimastigotes. After 5 weeks of selection with 250 μg/ml G418 and 10 μg/ml blasticidin, *TcMICU1* gene replacement was verified by PCR using primers 12 and 13 (Table S1).

### Knockout of *TcMICU2*.

An sgRNA to target the sequence coding for the hypothetical TcMICU2 protein (TryTripDB ID TcCLB.510525.130) was amplified by PCR (primers 8 and 14 [Table S1]). Following the same strategy mentioned above for *TcMICU1*, we obtained the *TcMICU2*-sgRNA/Cas9/pTREX-n construct. The DNA donor cassette to induce homologous directed repair and replacement of *TcMICU2* was obtained by PCR using a set of ultramers designed as described above (primers 15 and 16 [Table S1]). Gene disruption of *TcMICU2* was verified by PCR using primers 17 and 18 (Table S1).

### Southern blot analysis.

For Southern blot analysis of *TcMICU1*-KO cells, total genomic DNA was isolated from epimastigotes by phenol-chloroform extraction, digested with PvuII, separated on a 0.8% agarose gel, and transferred to nylon membrane and hybridized with a biotin-labeled fragment of 430 nt (*TcMICU1* [nt +655 to +1085]) obtained by PCR (Table S1, primers 19 and 20) using a cloned *TcMICU1* gene as the template. The probe was labeled using the North2South biotin random prime labeling kit. Hybridization, posthybridization washes, and detection were performed with the North2South chemiluminescent hybridization and detection kit, following the manufacturer’s recommendations. Signal detection was performed using the UVItec Alliance gel documentation system (UVItec, Cambridge, United Kingdom).

Alternatively, to check *TcMICU2*-KO by Southern blotting, approximately 25 μg of gDNA was digested with HindIII enzyme and separated on a 0.8% agarose gel. Restriction fragments were transferred to nylon membrane and hybridized with a biotin-labeled probe, which spans the 436 nt (*TcMICU2* [nt +195 to +631]) obtained by PCR (Table S1, primers 21 and 22) using the cloned *TcMICU2* gene as the template. Probe labeling, hybridization, and detection were performed as described for *TcMICU1*-KO Southern blot analysis.

Another strategy was the use of probes of 568 nt that recognize the *Bsd* gene. Twenty-five micrograms of gDNA from control, *TcMICU1*-KO, and *TcMICU2*-KO epimastigotes was digested with HindIII enzyme and resolved on a 0.8% agarose gel. Restriction fragments were transferred to nylon membrane and hybridized with a biotin-labeled probe corresponding to the full-length *Bsd* gene cloned in the pGEM-T Easy plasmid (Promega), amplified by PCR, and labeled using the North2South biotin random prime labeling kit. Hybridization, posthybridization washes, and detection were performed with North2South chemiluminescent hybridization and detection kit, following the manufacturer’s recommendations. Signal detection was performed using the UVItec Alliance gel documentation system (UVItec, Cambridge, United Kingdom).

### Cell transfections.

Transfections were performed as previously described (31). Briefly, T. cruzi Y strain epimastigotes (4 × 10^7^ cells) were washed with phosphate-buffered saline (PBS) at pH 7.4 at room temperature (RT) and transfected in ice-cold CytoMix (120 mM KCl, 0.15 mM CaCl_2_, 10 mM K_2_HPO_4_, 25 mM HEPES, 2 mM EDTA, 5 mM MgCl_2_ at pH 7.6) containing 25 μg of each plasmid construct in 4-mm electroporation cuvettes with three pulses (1,500 V, 25 μF) delivered by a Gene Pulser Xcell electroporation system (Bio-Rad). Stable cell lines were established and maintained under drug selection with appropriate antibiotic(s) (250 μg/ml G418, 10 μg/ml blasticidin, or 5 μg/ml puromycin). Transfectant epimastigotes were cultured in LIT medium supplemented with 20% heat-inactivated FBS until stable cell lines were obtained.

### Western blot analysis.

Western blot analyses were performed as previously described (31, 69, 70). Briefly, parental and mutant cell lines were harvested separately. Parasites were washed twice in PBS and resuspended in radioimmunoprecipitation assay (RIPA) buffer (150 mM NaCl, 20 mM Tris-HCl at pH 7.5, 1 mM EDTA, 1% SDS, 0.1% Triton X-100) plus a mammalian cell protease inhibitor mixture (diluted 1:250), 1 mM phenylmethylsulfonyl fluoride, 2.5 mM tosyl phenylalanyl chloromethyl ketone (TPCK), 100 μM N-(*trans*-epoxysuccinyl)-l-leucine 4-guanidinobutylamide (E64), and Benzonase nuclease (25 U/ml of culture). For Western blots to detect the phosphorylated PDH-E1α subunit, phosphatase inhibitors (1 mM Na_3_VO_4_ and 1 mM NaF) were also added to the RIPA buffer. The cells were then incubated for 1 h on ice, and the protein concentration was determined by BCA protein assay. Thirty micrograms of protein from each cell lysate was mixed with 6× Laemmli sample buffer (125 mM Tris-HCl at pH 7, 10% [wt/vol] β-mercaptoethanol, 20% [vol/vol] glycerol, 4.0% [wt/vol] SDS, 4.0% [wt/vol] bromophenol blue) before application to 10% SDS–polyacrylamide gels. When nonreducing conditions were required, protein extracts were mixed with sample buffer lacking β-mercaptoethanol. Separated proteins were transferred onto nitrocellulose membranes with a Bio-Rad Trans-blot apparatus. Membranes were blocked with 5% nonfat dried skim milk in PBS-T (PBS containing 0.1% [vol/vol] Tween 20) overnight at 4°C. Next, blots were incubated for 1 h at room temperature (RT) with a primary antibody: i.e., monoclonal anti-HA (1:5,000), rabbit polyclonal anti PDH-E1α (phospho-Ser-293) (1:1,000), and monoclonal antitubulin (1:40,000). After three washes with PBS-T, blots were incubated with the secondary antibody (goat anti-mouse IgG diluted 1:10,000 or goat anti-rabbit IgG HRP-conjugated antibody diluted 1:15,000). Membranes were washed three times with PBS-T, and Western blot images were obtained and processed with a C-DiGit blot scanner (LI-COR Biosciences).

### Immunofluorescence microscopy.

Cells were washed with PBS and fixed with 4% paraformaldehyde in PBS for 1 h at RT. To determine mitochondrial localization of proteins, epimastigotes were incubated with 100 nM MitoTracker deep red FM for 30 min at 28°C in culture medium before the fixing procedure. Cells could adhere to poly-l-lysine-coated coverslips and then were permeabilized for 5 min with 0.1% Triton X-100. Permeabilized cells were blocked overnight at 4°C with PBS containing 3% BSA, 1% fish gelatin, 50 mM NH_4_Cl, and 5% goat serum. Next, cells were incubated with a monoclonal anti-HA (1:5,000) or rabbit anti-TcATG8.1 (1:100) diluted in 1% BSA in PBS (pH 8.0) for 1 h at RT. Cells were washed three times with 1% BSA in PBS (pH 8.0), and then cells were incubated for 1 h at RT in the dark with Alexa Fluor 488-conjugated goat anti-mouse or Alexa Fluor 546-conjugated goat anti-rabbit secondary antibodies (1:1,000). Then, cells were washed and mounted on slides using Fluoromount-G mounting medium containing 5 μg/ml of 2-(4-aminophenyl)-1-indole-6-carboxamidine (DAPI) to stain DNA. Controls were treated as described above, but in the absence of a primary antibody. Differential interference contrast and fluorescence optical images were captured with a Leica TCS SP5 II confocal microscope, with a 100× objective (1.44 aperture) under nonsaturating conditions, that uses photomultiplier tubes (PMTs) for detection of emission, and LAS AF software (Leica, Wetzlar, Germany) for acquisition and processing of digital images.

### Ca^2+^ uptake by digitonin-permeabilized T. cruzi epimastigotes.

Cells were collected by centrifugation at 1,000 × *g* for 7 min and washed twice with buffer A with glucose (BAG: 116 mM NaCl, 5.4 mM KCl, 0.8 mM MgSO_4_, 5.5 mM d-glucose, and 50 mM HEPES at pH 7.0). Epimastigotes were resuspended to a final density of 1 × 10^9^ cells/ml in BAG and kept on ice. Before each experiment, a 50-μl aliquot of T. cruzi epimastigotes (5 × 10^7^ cells) was added to the reaction buffer (125 mM sucrose, 65 mM KCl, 10 mM HEPES-KOH buffer at pH 7.2, 1 mM MgCl_2_, 2.5 mM potassium phosphate [1.95 ml]) containing 5 mM succinate, 50 μM EGTA, and 0.5 μM fluorescent cell-impermeable Ca^2+^ indicator Fluo-4 (high affinity, used for low-[Ca^2+^]_ext_ conditions) or 0.5 μM Calcium Green-5N (low affinity, used for high-[Ca^2+^]_ext_ conditions). Mitochondrial Ca^2+^ uptake was initiated by the addition of different concentrations of free calcium, which were calculated using the software Maxchelator Calculator v1.2 (https://somapp.ucdmc.ucdavis.edu/pharmacology/bers/maxchelator/CaEGTA-NIST.htm). This calcium addition was followed by 50 μM digitonin and 4 μM FCCP. Fluorescence changes were monitored in an F-4500 or F-7000 fluorescence spectrophotometer (Hitachi) with excitation at 494 nm and emission at 520 nm using Fluo-4 or excitation at 506 nm and emission at 532 nm using Calcium Green-5N. The relative of calcium uptake was calculated as the absolute value of the slope of the linear regression fit in the linear range of the fluorescent signal (250 to 300 s using Calcium Green-5N and 150 to 200 s using Fluo-4). The relative rates of Ca^2+^ uptake were normalized for the control strains. The hyperbolic equation [Ca^2+^] = *K_d_* × [(*F* − *F*_min_)/(*F*_max_ − *F*)] was used to convert the raw fluorescence readings measured during mitochondrial Ca^2+^ transport assays into Ca^2+^ concentration levels, where *K_d_* is the dissociation constant, *F* is any given fluorescence value, *F*_min_ is the lowest fluorescence reading after addition of 0.5 mM EGTA, and *F*_max_ is the maximal fluorescence obtained after two sequential additions of 1 mM CaCl_2_. These additions were performed at the end of each trace. *K_d_* for Ca^2+^ indicator probes in our conditions was determined according Chweih et al. (71). Mitochondrial Ca^2+^ uptake rates were calculated as the first derivative of the absolute values of the slope by using the SLOPE Excel function for 20 points (250 to 270 s using Calcium Green-5N and 150 to 170 s using Fluo-4).

### Mitochondrial membrane potential.

Estimation of mitochondrial membrane potential *in situ* was done spectrofluorometrically using the indicator dye safranin O, as described previously (31, 72). Briefly, T. cruzi epimastigotes (5 × 10^7^ cells) were incubated at 28°C in reaction buffer (125 mM sucrose, 65 mM KCl, 10 mM HEPES-KOH buffer at pH 7.2, 1 mM MgCl_2_, 2.5 mM potassium phosphate [1.95 ml]) containing 5 mM succinate, 0.2% BSA, 50 μM EGTA, and 5 μM safranin O, and the reaction was started with digitonin (50 μM). ADP (250 μM), carboxyatractyloside (1.5 μM), and FCCP (4 μM) were added to the medium at different time points. Fluorescence changes were monitored using the Hitachi F-4500 or F-7000 spectrofluorometer (excitation of 495 nm and emission of 586 nm).

### Cellular respiration.

The OCR of digitonin-permeabilized epimastigotes was measured using a high-resolution respirometer (Oroboros Oxygraph-2k; Oroboros Instruments GmbH, Innsbruck, Austria) with DatLab 4 software for data acquisition and analysis. The equipment was calibrated as reported by its manufacturer. Cells (1 × 10^8^) were incubated at 28°C in a 2-ml chamber containing 125 mM sucrose, 65 mM KCl, 10 mM HEPES-KOH (pH 7.2), 2.5 mM K_2_PO_4_, 1 mM MgCl_2_, 50 μM EGTA, 5 mM succinate, and 25 μM digitonin. OCR was calculated as the negative time derivative of the oxygen concentration measured in the close respirometer chambers and expressed per milligram of protein. Data were recorded at 2-s intervals, and 10 data points were used to calculate the slope of the OCR plot through a polynomial fit with DatLab 4 software, as described previously (73).

### Citrate synthase activity.

Citrate synthase activity was measured using a previously described protocol (74) adapted to trypanosomes (31). Briefly, the conversion of oxaloacetate and acetyl coenzyme A (acetyl-CoA) to citrate and SH-CoA was monitored by quantification of the colorimetric product thionitrobenzoic acid (75). T. cruzi epimastigotes in early exponential phase (∼1 × 10^8^ cells) were washed twice with PBS and incubated in lysis buffer (10 mM Tris-HCl at pH 7.4, 1 mM EDTA, 0.1% Triton X-100, and 25 U of Benzonase nuclease) for 10 min on ice. Then, proteins were quantified by BCA protein assay, and 260-μl reactions were set up in buffer containing 5 μg protein, 250 μM oxaloacetate, 50 μM acetyl-CoA, 100 μM 5,5′-dithio-bis (2-nitrobenzoic acid), and 10 mM Tris-HCl (pH 8.0). The increase in absorbance at 412 nm was monitored for 20 min at 28°C using a microplate reader (PowerWave XS 2, BioTek Instruments, Winooski, VT). *V*_max_ values were normalized taking the scrambled cell line as the reference value.

### Autophagy assay.

Expression of the TcAtg8.1 autophagy marker and autophagosome formation in T. cruzi epimastigotes grown in LIT medium and under starvation conditions were estimated by immunofluorescence analyses using anti-TcATG8.1 antibody as described previously (43). For starvation induction, mid-log-phase parasites were washed twice with PBS, resuspended in the same buffer at a concentration of 5 × 10^7^ cells/ml, and incubated for 16 h at 28°C as described previously (43).

### Adenine nucleotide levels.

Control (transfected with scrambled sgRNA), *TcMICU1*-KO, and *TcMICU2*-KO epimastigotes were harvested and washed once with buffer A (116 mM NaCl, 5.4 mM KCl, 0.8 mM MgSO_4_, and 50 mM HEPES at pH 7.0). After being washed, 1 × 10^8^ cells per treatment were centrifuged and resuspended in 100 μl of buffer A and then lysed on ice for 30 min by addition of 150 μl of 0.5 M HClO_4_. The lysates were neutralized (pH 6.5) by addition of 60 μl of 0.72 M KOH–0.6 M KHCO_3_. Samples were centrifuged at 1,000 × *g* for 5 min, and the supernatant was separated for adenine nucleotide determination. ATP, ADP, and AMP in extracted samples were quantified by a luciferin-luciferase bioluminescence assay in a luminometer as described previously (31), with some modifications. We used an ATP determination kit (Invitrogen) according to the manufacturer’s instructions with adenylate kinase and/or nucleoside-diphosphate kinase (NDK [Sigma]). To determine the amount of adenine nucleotides, four measurements were taken of three different reactions for each sample by endpoint determination of the ATP concentration: one reaction without addition of any ATP-generating enzyme (for ATP), another reaction adding NDK (for ATP + ADP), and a third reaction adding both adenylate kinase and NDK (for ATP + ADP + AMP). The amount of ADP was obtained by subtracting the ATP value from the ATP + ADP value and the amount of AMP was calculated from the difference between the ATP + ADP + AMP content and the ATP + ADP content.

### Metacyclogenesis.

For metacyclogenesis, we followed the protocol described by Bourguignon et al. (76) with minor modifications. Epimastigotes were obtained after 4 days in LIT medium and submitted to a stress by incubation for 2 h at room temperature in triatomine artificial urine (TAU) medium (190 mM NaCl, 17 mM KCl, 2 mM MgCl_2_, 2 mM CaCl_2_, 0.035% sodium bicarbonate, and 8 mM phosphate at pH 6.9). After this stress, parasites were incubated for 96 h in TAU 3AAG medium (which consists of the above-described TAU medium supplemented with 10 mM l-proline, 50 mM sodium l-glutamate, 2 mM sodium l-aspartate, and 10 mM glucose). To increase the number of metacyclic forms to infect Vero cells, the contents of the flask were collected and resuspended in medium containing fresh fetal bovine serum and incubated at 37°C for 20 h. The complement in the FBS kills epimastigotes, while metacyclic trypomastigotes survive. Samples were harvested from the TAU 3AAG plus FBS-containing medium at days 5 and 10 of cultivation.

### *In vitro* infection assay.

Gamma-irradiated (2,000 rads) Vero cells (4.5 × 10^5^ cells) were plated onto sterile coverslips in a 12-well plate and incubated overnight at 35°C in 7% CO_2_ in RPMI medium plus 10% fresh fetal bovine serum. Tissue culture-derived trypomastigote collections were incubated at 4°C overnight to allow amastigotes to settle from swimming trypomastigotes. Trypomastigotes from the supernatants of these collections were counted and used to infect the coverslips at a ratio of 50 parasites to 1 host cell. At 4 h postinfection, coverslips were washed extensively with Hanks’ solution, followed by phosphate-buffered saline (PBS) at pH 7.4 to remove any extracellular parasites. Coverslips were fixed immediately in 4% paraformaldehyde in PBS (pH 7.4) at 4°C for 30 min. Coverslips were washed once with PBS and mounted onto glass slides in Fluoromount-G containing 15 μg/ml of 2-(4-aminophenyl)-1H-indole-6-carboxamidine (DAPI), which stains host and parasite DNA. Coverslips were viewed on an Olympus BX60 microscope to quantify the number of host cells that contained intracellular parasites and the number of intracellular parasites per cell in randomly selected fields. Three hundred host cells were counted per sample in three independent experiments. To quantify amastigote replication, the following modifications were used: host cells were infected at a ratio of 10 parasites to 1 host cell, and coverslips were allowed to incubate for 48 h postinfection at 35°C in 7% CO_2_, prior to fixation and DAPI staining.

### Statistical analysis.

Statistical analyses were performed with GraphPad Prism software version 7.4 (GraphPad, La Jolla, CA). Reported values are means ± standard deviation (SD) from *n* biological experiments, as indicated in the figure legends. The level of significance was evaluated by Student's *t* test for comparisons between two cell lines, one-way analysis of variance (ANOVA) for comparisons between more than two cell lines, and two-way ANOVA with multiple-comparison tests for analyses of grouped data.

10.1128/mBio.00348-19.6 FIG S6 Complete Western blots shown in this work. Download FIG S6, TIF file, 1.6 MB. Copyright © 2019 Bertolini et al. 2019 Bertolini et al. This content is distributed under the terms of the Creative Commons Attribution 4.0 International license.
