# Supplementary figures and images for: MICU1 and MICU2 Play an Essential Role in Mitochondrial Ca2+ Uptake, Growth, and Infectivity of the Human Pathogen Trypanosoma cruzi
Source: mBio. 2019 May 7;10(3):e00348-19. doi: 10.1128/mBio.00348-19 (PMC6509184; doi:10.1128/mBio.00348-19)

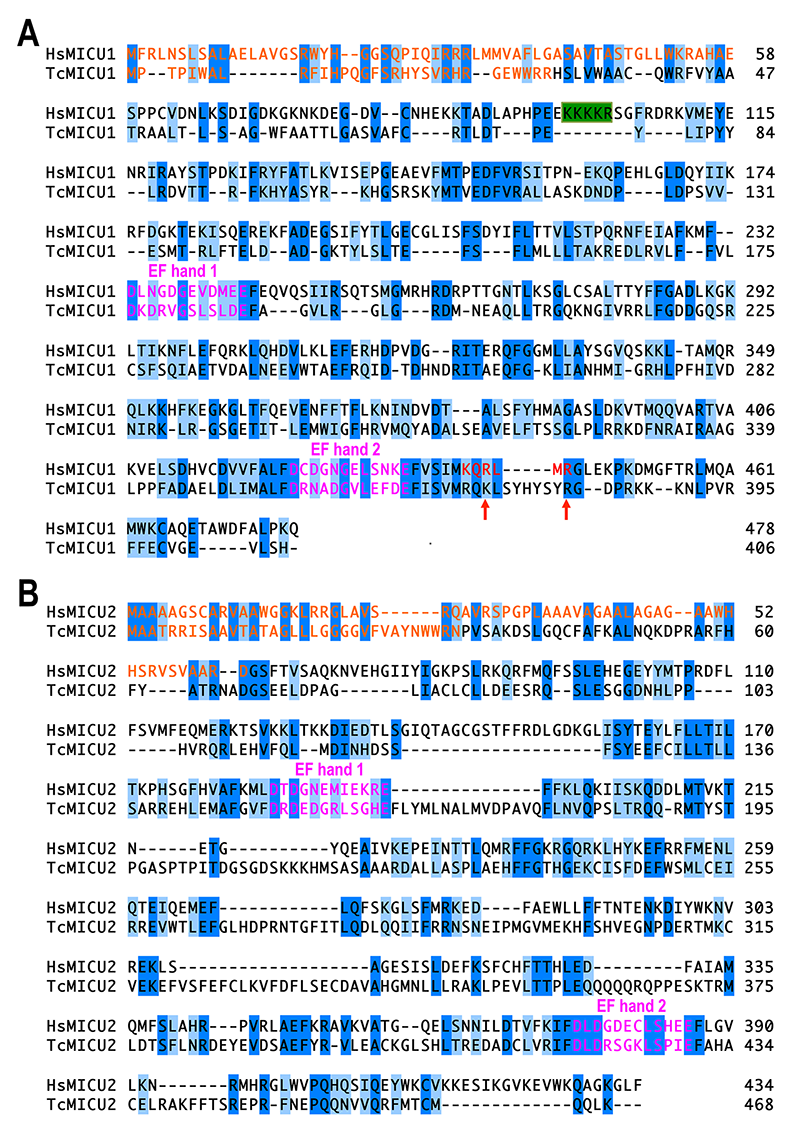

Supplement: FIG S1 [file mBio.00348-19-sf001.tif]

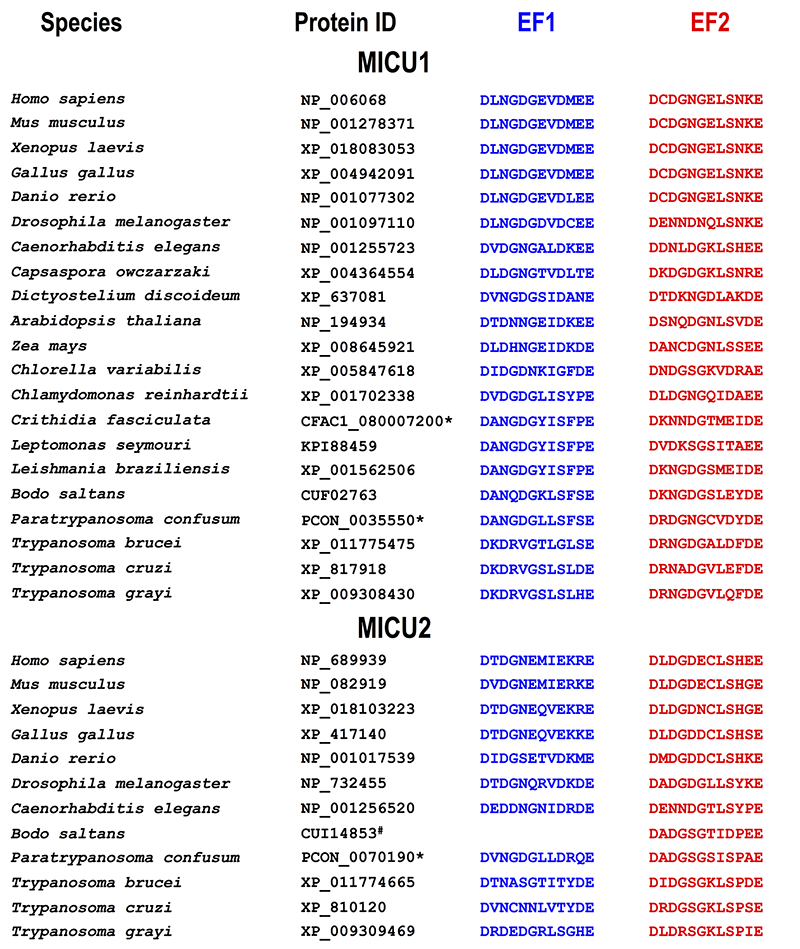

Supplement: FIG S2 [file mBio.00348-19-sf002.tif]

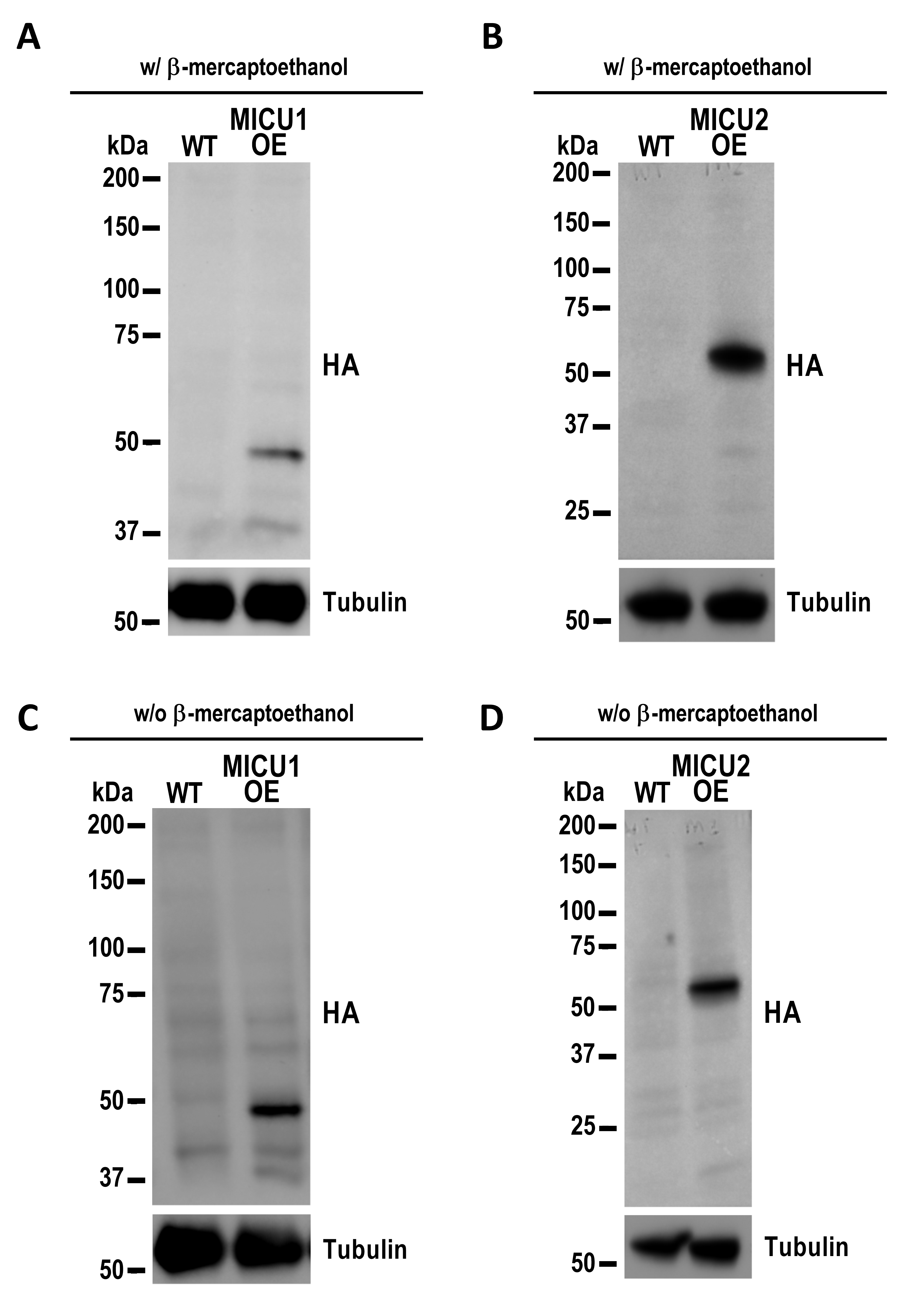

Supplement: FIG S3 [file mBio.00348-19-sf003.tif]

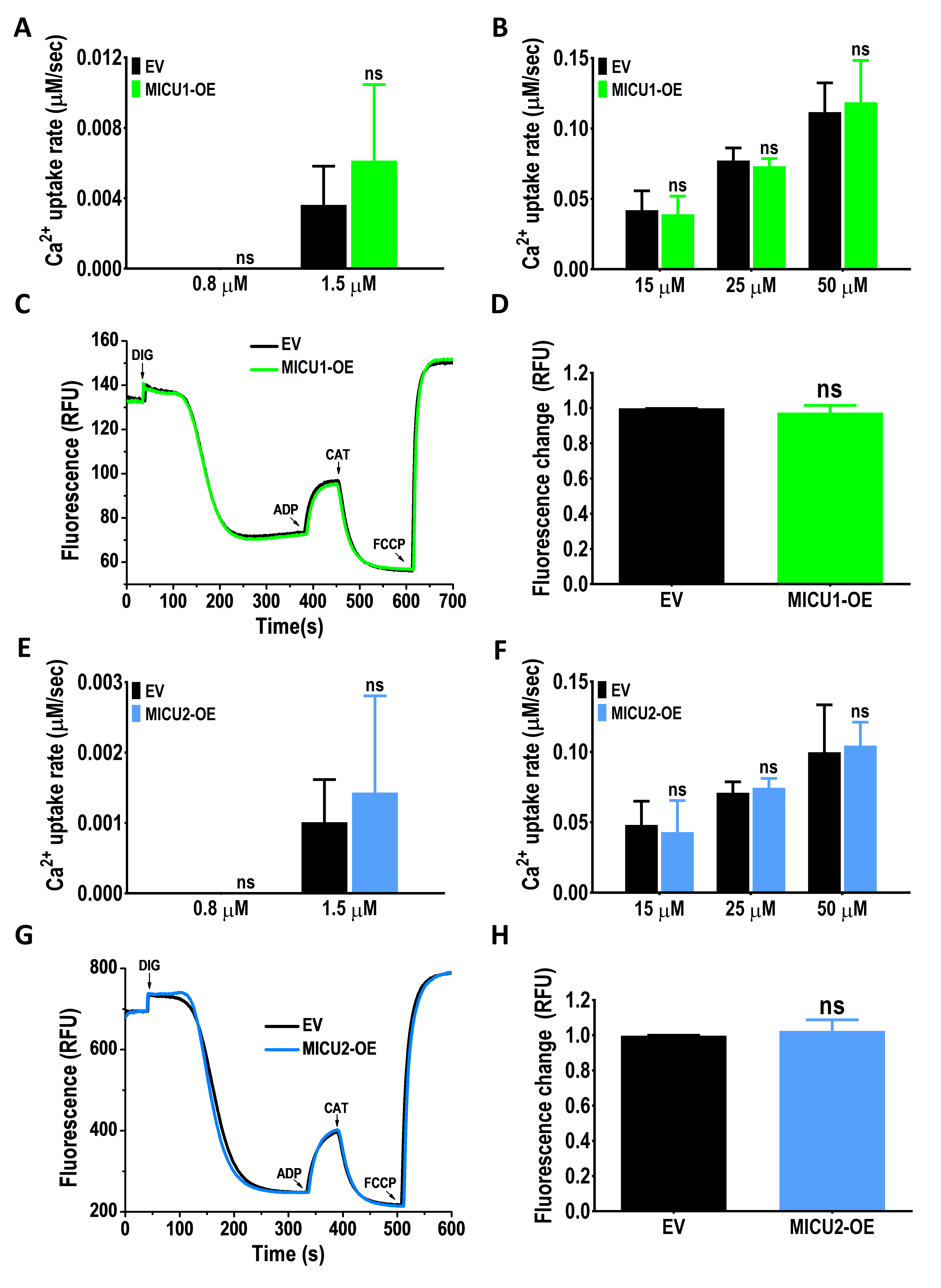

Supplement: FIG S4 [file mBio.00348-19-sf004.tif]

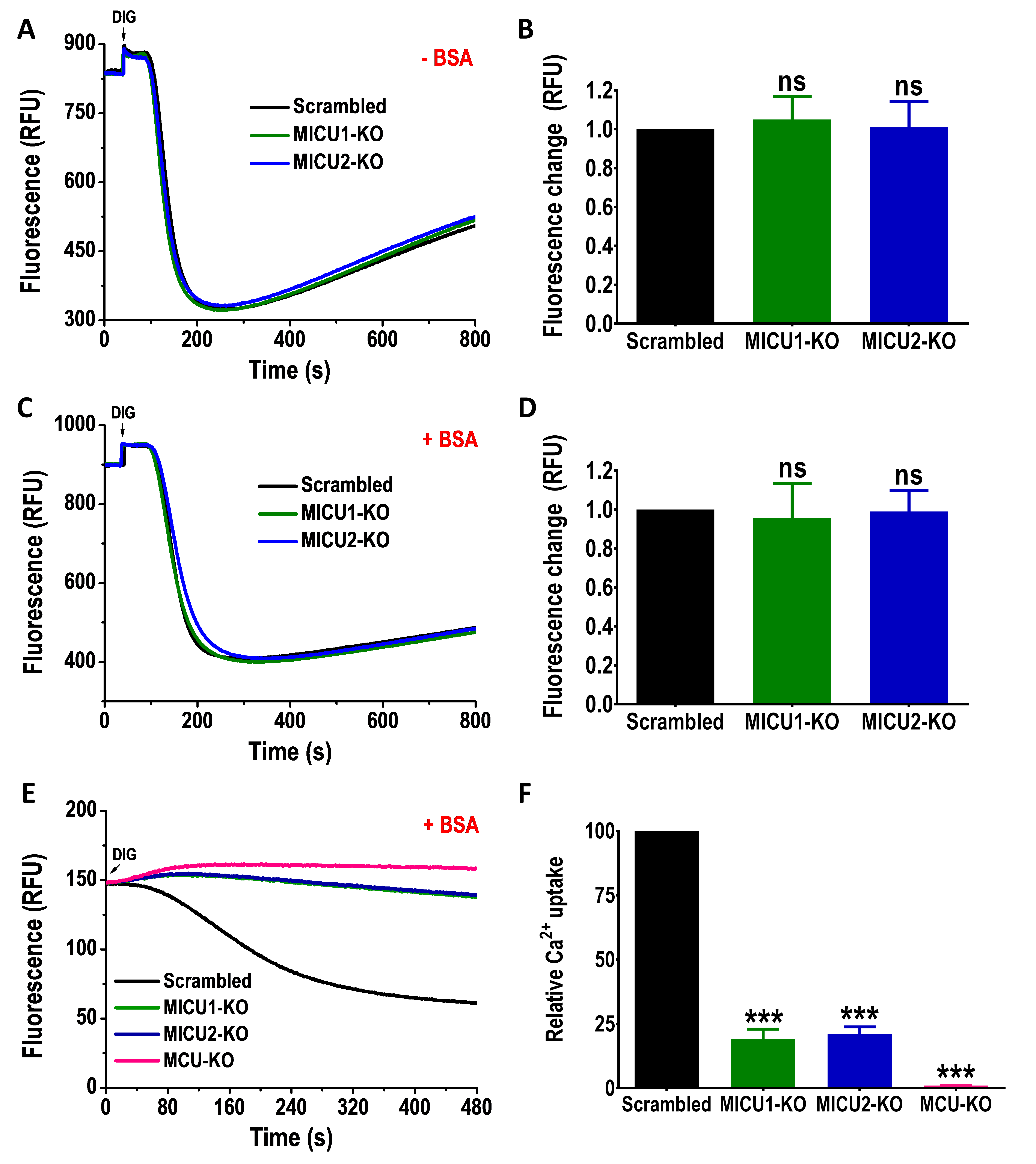

Supplement: FIG S5 [file mBio.00348-19-sf005.tif]

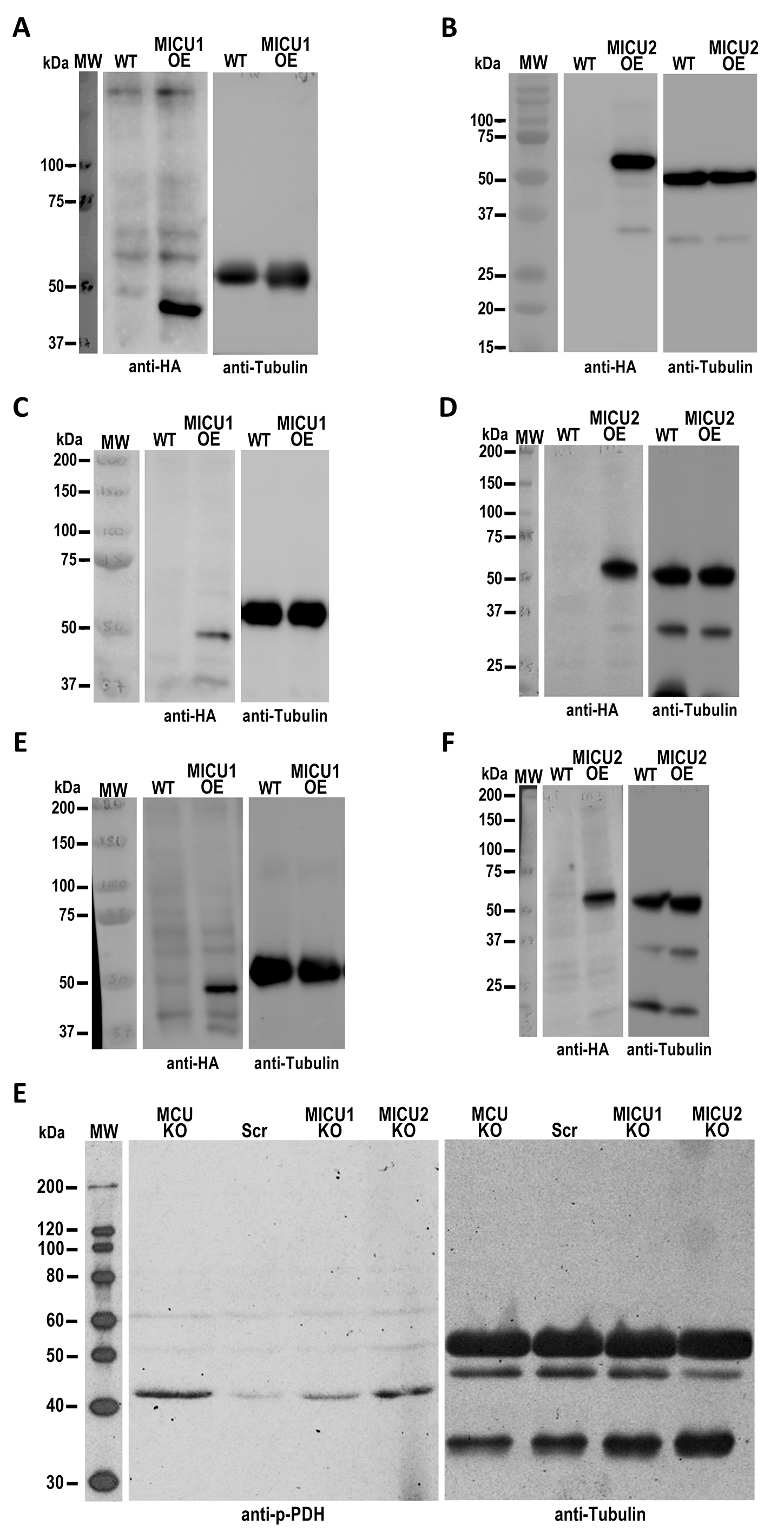

Supplement: FIG S6 [file mBio.00348-19-sf006.tif]
