# Supplementary material for: MICU1 and MICU2 Play an Essential Role in Mitochondrial Ca2+ Uptake, Growth, and Infectivity of the Human Pathogen Trypanosoma cruzi
Source: mBio. 2019 May 7;10(3):e00348-19. doi: 10.1128/mBio.00348-19 (PMC6509184; doi:10.1128/mBio.00348-19)
Supplement: TABLE S1 [file mBio.00348-19-st001.docx]

**Table S1.** Oligonucleotides used in this work.

| **N°** | **Primer name** | **Sequence (5’ → 3’)** |
| --- | --- | --- |
| **1** | **FwTcMICU1_XbaI** | GATC*TCTAGA*ATGCCTACTCCGATTTGGG |
| **2** | **Rv_MICU1_HA_P2A** | ACGTCGTAGGGATAATGAGACAACACTTCACC |
| **3** | **Fw_MICU1_2XHA_P2HA** | TTGTCTCATTATCCCTACGACGTGCCTGATTATGCATACCCATATGATGTCCCCGACTATGCCGGATCCGG |
| **4** | **Rv_pC034_BSD_XhoI** | AGTT*CTCGAG*TTAGCCCTCCCACACATAAC |
| **5** | **FwTcMICU2_EcoRI** | GATC*GAATTC*ATGGCTGCAACAAGGCGCA |
| **6** | **RvTcMICU2-2xHA_XhoI** | CTAT*CTCGAG*TCACGCGTAGTCCGGCACGTCGTACGGGTACGCGTAGTCCGGCACGTCGTACGGGTACGCGT |
| **7** | **FwTcMICU1_sgRNA10** | GATC*GGATCC***GCATTATTCCGTGCGTCACC**GTTTTAGAGCTAGAAATAGC |
| **8** | **RvSgRNA** | CAGTGGATCCAAAAAAGCACCGACTCGGTG |
| **9** | **Rv_HXI** | TAATTTCGCTTTCGTGCGTG |
| **10** | **Fw_TcMICU1_donor** | **ATGTTGCTTTTACACTTTGTTTGTTTGTTTTGTTTTGTTTGGGTTTCTCCTTCGAGGTGCTGCCGTGTTTAGGATCCGGAATTCCATTGCGTGGCGCCGC**ATGGCCAAGCCTTTGTCTCA |
| **11** | **Rv_TcMICU1_donor** | **CCAGCAGACACACGGAAAGAAAAGAGGGGAGGGAAACAACAAAAAACGGAAAGCAGGAGGTAAATAGAGAATACATGCAACACAAAGGTAAAATGCCCAA**TTAGCCCTCCCACACATAAC |
| **12** | **Fw_MICU1-KO_check** | CTTGTGTGCCGCATATGTGTG |
| **13** | **Rv_MICU1-KO_check** | GAGCGATCTGTACACGACG |
| **14** | **FwTcMICU2_sgRNA14** | GATC*GGATCC***GCCTTTGGGGTCTTTGACAG**GTTTTAGAGCTAGAAATAGC |
| **15** | **Fw_TcMICU2_donor** | **CCTCTAATGTCTCATGGCTACTCAATGACGTTCCCTCTCATTATTTGTTTATCCAAACACCACTATTTCCTGTATTTGTTACGATGCCAAGGAACCACAC**ATGGCCAAGCCTTTGTCTCA |
| **16** | **Rv_TcMICU2_donor** | **GAGTAAGGGAGAAATGAGACAACAACAATAACAACATAAAAAACATGATCGACGATCATTCCGTGAGCCTGAAAATAAAAATCACTGCGAATGCGTTCGA**TTAGCCCTCCCACACATAAC |
| **17** | **Fw_MICU2-KO_check** | GGGGGGCGAAAATTTGTGTTG |
| **18** | **Rv_MICU2-KO_check** | CATAACGTAGAGCAGAGACAATC |
| **19** | **Fw_MICU1-probe** | TGGTGACGATGGACAAAGC |
| **20** | **Rv_MICU1-probe** | ACGCCATCTGCATTACGGTC |
| **21** | **Fw_MICU2-probe** | AATGCAGATGGGAGTGAGG |
| **22** | **Rv_MICU2-probe** | TTTGAATCCCCTGAGCCG |

Bold uppercase: specific protospacer; italic uppercase: restriction site; bold underlined uppercase: gene-specific homologous region; italic lower case: mismatch nucleotides
